# Correlations and nonlinear partition of nonionic organic compounds by humus-like substances humificated from rice straw

**DOI:** 10.1038/s41598-019-51406-3

**Published:** 2019-10-22

**Authors:** Liufen Ren, Daohui Lin, Kun Yang

**Affiliations:** 1 Department of Environmental Science, Zhejiang University, Hangzhou, 310058 China; 2 Key Laboratory of Environmental Pollution and Ecological Health of Ministry of Education, Hangzhou, 310058 China; 3 Zhejiang Provincial Key Laboratory of Organic Pollution Process and Control, Hangzhou, 310058 China

**Keywords:** Environmental sciences, Environmental chemistry

## Abstract

The debate on whether the nonlinear sorption of nonionic organic compounds (NOCs) by soil organic matter (SOM) is captured by nonlinear partition or adsorption has been going on for decades because the used SOM samples are complex mixtures from various precursors with varied humification degrees in natural environment. Therefore, in this study, hydrothermal method was employed to prepare humus-like substances from a sole precursor (i.e., rice straw) with various humification degrees for nonlinear sorption of 25 aromatic compounds, then to have an insight into the underlying mechanisms of the nonlinear sorption of NOCs by SOM. It was observed that the increasing humification degree of humus-like substances, i.e., decreasing in the polarity ((O + N)/C) and increasing in the aromaticity, result in the increase of isotherm nonlinearity and sorption capacity/affinity of NOCs. Sorption capacity of NOCs, obtained by isotherm fitting using Dubinin-Astakhov (DA) model and Dual-Mode (DM) model, are positively correlated with their solubility in water and octanol, indicating the nonlinear sorption could be captured by nonlinear partition mechanism. Specific interactions including hydrogen-bonding interaction and *π*-*π* interaction between aromatic structures of humus-like substances and organic molecules could be responsible for the nonlinear partition and the increase of sorption affinity with the enhancement of humification degree. These obtained correlations are valuable for understanding the underlying mechanisms of nonlinear sorption and elucidating the transport of NOCs in the environment.

## Introduction

Nonionic organic compounds (NOCs) such as phenanthrene, pyrene, 4-chloroaniline are widely released into the environment and observed in soils with significant concentrations^1–5^. For example, 500 mg/kg phenanthrene in soil from Toledo (Spain) was detected^2^. Most NOCs are persistent organic pollutants, with toxic, carcinogenic and mutagenic properties^3,4^. Therefore, environmental risks of NOCs arouse increasing attentions. Nonlinear sorption, companied commonly with competitive sorption^6–8^ and irreversible sorption^9,10^ of NOCs by soil is a significant behavior that controlling their transport, fate and bioavailability in environment^7,11^. Thus, understanding the nonlinear sorption mechanism is crucial for assessing the environmental risks of NOCs. In addition to soil mineral and small amount of high-surface-area carbonaceous material (HSACM) such as charcoal or soot in soil, soil organic matter (SOM) was suggested to be the primary soil component responsible for the nonlinear sorption^5–7,12,13^. Therefore, nonlinear isotherms were observed for nonpolar compounds sorption by black carbon-free SOM^12^. We also observed that nonlinear sorption capacity of both polar 2,4-dichorophenol and nonpolar phenanthrene is largely dependent on the SOM content in soil^11,13^.

SOM is well known as a complex mixture of partially or incompletely decomposed biopolymer of plant and animal origins, having diverse physicochemical characteristics^14,15^. Thus, the significant differences of chemical composition, structures, and conformation among SOM were widely employed to account for the differences in nonlinear sorption of NOCs, but there are several debates for decades. For example, it was suggested that SOM consists of two typical domains, characterized as “soft carbon” and “hard carbon”^10,14^ or as “rubbery state” and “glassy state”^9,10,14^, respectively. Sorption by hard carbon (i.e., glassy state domain) is interpreted by the adsorption mechanism and employed to be responsible for nonlinear sorption, while sorption by soft carbon (i.e., rubbery state domain) is interpreted by the partition mechanism with linear isotherms^9,10,14^. The term “sorption” used here to denote the uptake of a solute without reference to a specific mechanism, i.e., “sorption” means that the mechanism could be adsorption, partition, or both of them^5,16^. Meanwhile, Spurlock and Biggar^17^ suggested that the nonlinear sorption could be attributed to the nonlinear partition of NOCs into SOM because NOCs, especially for the polar ones, can form specific interactions such as H-bonding with the limited active sites in SOM. Nonlinear partition is a partition behavior rather than adsorption behavior. Moreover, different from the normal partition behavior with linear isotherm, nonlinear partition is with isotherm nonlinearity^17,18^. Linear partition is dominated by van der Waals force alone, while the nonlinear partition and isotherm nonlinearity could be interpreted by the additional specific interactions such as H-bonding to van der Waals force^17,18^. Nonlinear partition has been employed to interpret the nonlinear sorption of polar NOCs by SOM in later studies^8,19,20^. The importance of aromaticity or aliphaticity as sorption domain was also debated^21^. Using ^13^C NMR for characterization of SOM, some researchers observed that both the nonlinear sorption capacity and the isotherm nonlinearity are positively proportional to the aromatic carbon content of SOM^22–24^, some observed that aliphatic-rich SOM has higher sorption capacity^25,26^, while other researchers observed that neither aromaticity nor aliphaticity of SOM could be directly related to the nonlinear sorption by plotting a large data set^27^. Moreover, negative relationship between isotherm nonlinearity of NOCs and the polarity ((O + N)/C) of SOM was reported^24,28–31^, but not observed in other studies^32^. A possible reason for these debates is the complex nature of SOM^27,33^. SOM from different sites has various precursors (i.e., plants and animals) and humification degrees due to the different temperature, weathering, diagenesis age, and so on^15,27^. Even within a site, the precursors and humification degrees of SOM are various^30^. Therefore, characteristics of SOM are commonly varied from site to site and from sample to sample within a site^15,27,30,31^.

For sorption of NOCs by SOM, until now, most researchers used SOM from different sites, different depths in a site, or sequentially extracted from a soil^6,7,11–33^. These used SOMs have various precursors and varied humification degrees. Therefore, sorption studies using SOM from sole precursor, having relatively simple structure and homogeneous nature in general, may provide helpful information for the insight into nonlinear sorption of NOCs by SOM. However, in natural environment, it is impossible to get a SOM sample from a sole precursor with a given humification degree. Hydrothermal treatment is a technique that has been successfully employed to simulate natural humification process of biomass residues in a short time because not only the occurred dehydration, decarboxylation reactions are similar to the natural humification process, but also the characteristics of obtained humus-like substances, such as aromatization degree, elemental composition and oxygen- containing groups, are similar to the natural SOM samples^21,34^. Moreover, by controlling the treatment conditions such as temperature and time, humus-like substance samples with expected humification degree can be obtained from a sole biomass. Thus, rice straw, a common and abundant precursor of SOM, was selected to prepare humus-like substances using the hydrothermal method in this work. Sorption of 25 typical NOCs, including nitroaromatic compounds, phenolic compounds, aromatic amines and polycyclic aromatic hydrocarbons (PAHs), by rice straw and the prepared humus-like substances were investigated to relate structural or compositional variations of these humus-like substances with nonlinear sorption of NOCs. The observations and results obtained from this study could not only address the long-time debates on nonlinear sorption mechanism and correlations of NOCs with the properties of SOM, but also help to elucidate the transport of NOCs in the environment.

## Results and Discussion

### Isotherms and model fitting

Isotherms of 25 NOCs on rice straw (RS0) and 6 humus-like substances (RS4, RS8, RS24, RS48, RS96, RS144) are well fitted by both Dubinin-Astakhov (DA) model and Dual-Mode (DM) model (Figs S1–S6), as is indicated by the relative coefficients (*r*^2^) close to 1, high F values and low SDEV values (Tables S1 and S2). The fitted results of DA model parameters (i.e., log*Q*^0^, *E*, *b*) and DM model parameters (i.e., *K*_p_, Q^*^, *K*_*L*_) are listed in Tables S1 and S2, respectively. The isotherm nonlinearity of 25 NOCs by rice straw is insignificant (Figs S1–S6), indicated by the fitted values of *E* close to 5.71 and *b* close to 1^18^. This is also supported by the much lower Q^*^ (fitted by DM model in Table S2) than *Q*^0^ in Table S1. The isotherm nonlinearity of 25 NOCs by humus-like substances increased with humification time, indicated by the increased *E* (Table S1) and Q^*^ (Table S2).

### Correlations of nonlinear sorption capacity with properties of NOCs

**Figure 1:**
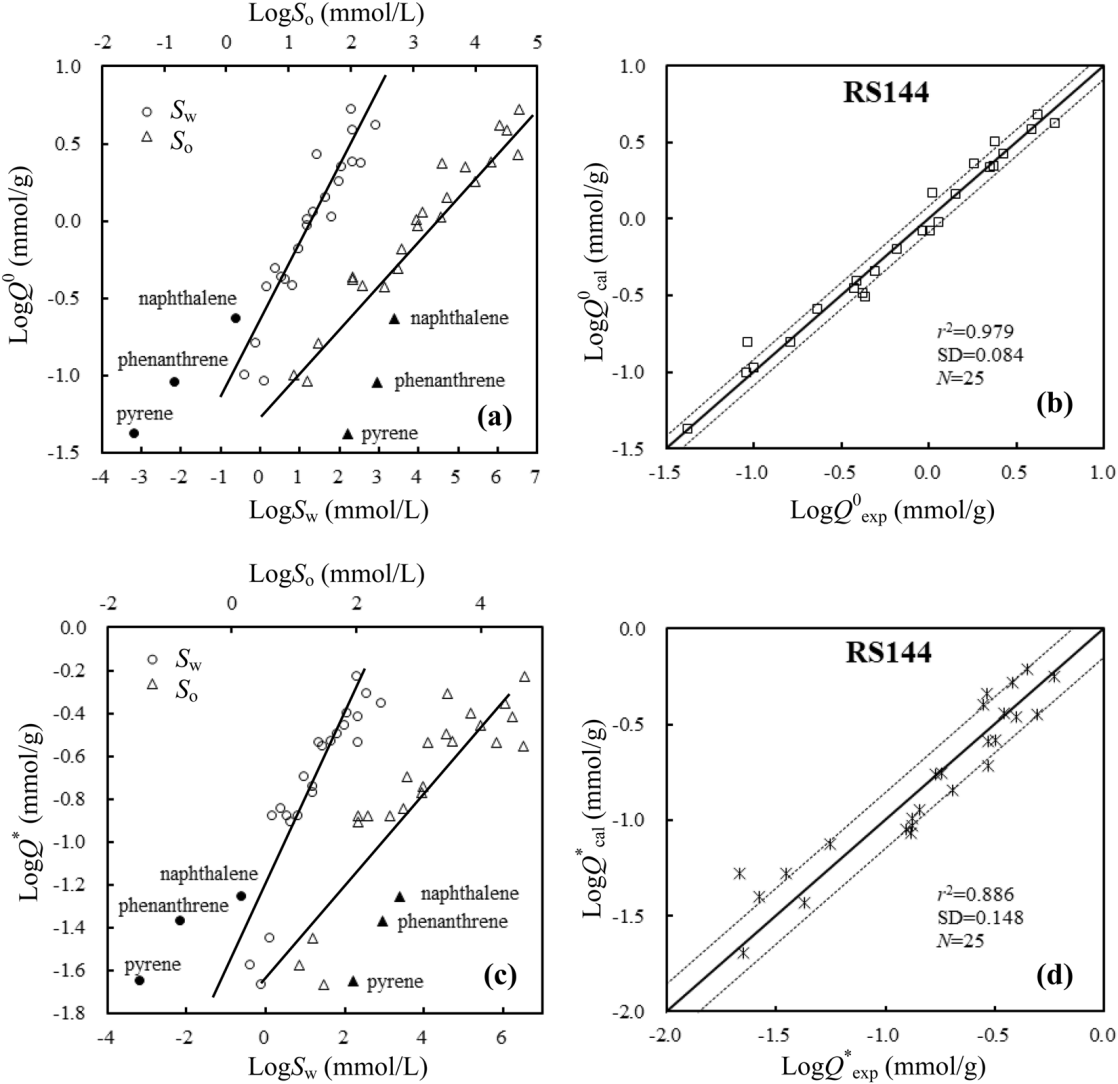
Linear relationships of log*Q*^0^ (**a**) and logQ^*^ (**c**) with log*S*_w_ or log*S*_o_ of 25 aromatic chemicals by RS144 (as an example of humus-like substances), as well as correlations of calculated sorption capacity log*Q*^0^_cal_ and logQ^*^_cal_ with experimental sorption capacity log*Q*^0^_exp_ (**b**) and logQ^*^_exp_ (**d**) by RS144, respectively. Solid lines are the reference line, y = x. Dotted lines are the standard deviation (SD) values from the reference line.

For a given humus-like substance or rice straw, positively logarithmic correlation between DA model fitted *Q*^0^ (Table S1) and solubility of 25 NOCs in water (*S*_w_) or octanol (*S*_o_, *S*_o_ = *S*_w_ × *K*_ow_, Table S3) was observed (Fig. 1a). These correlations, obtained in *S*_w_ and *S*_o_ range of about 6 orders and 4 orders of magnitude respectively, are consistent with the partitioning of NOCs into natural SOM or organic solvents such as octanol^5,35^, indicating that rice straw or humus-like substance acts as a partition medium and the nonlinear sorption of NOCs by rice straw or humus-like substance could be captured by the partition-like mechanism, i.e., nonlinear partition suggested by Spurlock and Biggar^17^. In the recent study, we observed that the positively linear relationship of sorption capacity (i.e., DA model fitted *Q*^0^) of NOCs with their solubility in water or octanol, but not the *K*_ow_, is the primary characteristic of not only the linear partition mechanism but also the nonlinear partition mechanism^35^. Deviations of PAHs (i.e., naphthalene, phenanthrene and pyrene) from these correlations, i.e., upward deviation from the log*Q*^0^ − log*S*_w_ fitting (Fig. 1a) and downward deviation from the log*Q*^0^ − log*S*_o_ fitting (Fig. 1a), were also observed. A possible reason is that PAHs are nonpolar while other compounds (i.e., nitroaromatic compounds, phenolic compounds, and aromatic amines) investigated in this study are polar. Therefore, in this work, an excellent and better relationship of log*Q*^0^ with both of log*S*_w_ and log*S*_o_ (Eq. 1), using multiple linear regression, was obtained to reduce the deviations of PAHs (listed in Table 1). The calculated sorption capacity log*Q*^0^_cal_ of 25 NOCs using Eq. 1 is well in accordance with the experimental sorption capacity log*Q*^0^_exp_ on rice straw or humus-like substances (Figs 1b and S7), indicating the observed multiple linear relationships are significant.

1 $$\log \,{Q}^{0}={\bf{A}}\times \,\log \,{S}_{{\rm{w}}}+{\bf{B}}\times \,\log \,{S}_{{\rm{o}}}+{\bf{C}}$$

where, **A**, **B** are the coefficients of log*S*_w_ and log*S*_o_, respectively. **C** is the intercept. The better fitting of Eq. 1 for log*Q*^0^ than that of log*Q*^0^ with log*S*_w_ or log*S*_o_ alone (Fig. 1a) implies that the ability of rice straw and humus-like substances to dissolved organic compounds could be stronger than water but weaker than octanol. Moreover, Eq. 1 indicates that nonlinear sorption capacity *Q*^0^ of organic compounds increases with not only *S*_w_ but also *S*_o_. The nonlinear partitioning mechanism of 25 NOCs into humus-like substances or rice straw is also supported by the good correlations of logQ^*^, i.e., the maximum nonlinear sorption capacity of nonlinear fraction separated from isotherms by DM model fitting, with log*S*_w_ or log*S*_o_ (Fig. 1c), especially the multiple linear relationship of logQ^*^ with log*S*_w_ and log*S*_o_ (Eq. 2, Table 2, Figs 1d and S8).

2 $$\log \,{Q}^{\ast }={\bf{D}}\times \,\log \,{S}_{w}+{\bf{G}}\times \,\log \,{S}_{{\rm{o}}}+{\bf{H}}$$

where **D**, **G** are the coefficients of log*S*_w_ and log*S*_o_, respectively. **H** is the intercept. Similar correlations of log*Q*_p_ (*Q*_p_ = *K*_p_ × *S*_w_), i.e., the partitioning capacity of linear partition fraction separated from isotherms by DM model fitting, with log*S*_w_ or log*S*_o_ (Fig. S9a), and the multiple linear relationship of log*Q*_p_ with log*S*_w_ and log*S*_o_ (Eq. 3, Table S4, Fig. S9b–h), were also observed.

3 $$\log \,{Q}_{{\rm{p}}}={\bf{J}}\times \,\log \,{{S}}_{{\rm{w}}}+{\bf{K}}\times \,\log \,{S}_{{\rm{o}}}+{\bf{L}}$$

where **J**, **K** are the coefficients of log*S*_w_ and log*S*_o_, respectively. **L** is the intercept. The correlations and relationships of sorption capacity with log*S*_w_ and log*S*_o_, observed for the nonlinear fraction (separated from isotherms by DM model fitting) in Eq. 2 and the DA model fitted sorption capacity in Eq. 1, are similar to the correlations for the linear partition fraction (separated from isotherms by DM model fitting) in Eq. 3, indicating that the nonlinear sorption of NOCs by rice straw or humus-like substances is captured by the nonlinear partition mechanism.

**Table 1:** Parameters A, B and C of Eq. 1 for rice straw (RS0) and humus-like substances (RS4, RS8, RS24, RS48, RS96, RS144).

Adsorbent	A	B	C	*r* ^2^	F	P
RS0	0.335 (±0.036)	0.368 (±0.049)	−2.26 (±0.13)	0.959	260	<0.001
RS4	0.251 (±0.022)	0.268 (±0.030)	−1.51 (±0.08)	0.972	382	<0.001
RS8	0.248 (±0.021)	0.259 (±0.028)	−1.46 (±0.08)	0.974	417	<0.001
RS24	0.221 (±0.019)	0.262 (±0.025)	−1.35 (±0.07)	0.976	452	<0.001
RS48	0.230 (±0.020)	0.265 (±0.027)	−1.27 (±0.07)	0.974	419	<0.001
RS96	0.236 (±0.017)	0.266 (±0.023)	−1.19 (±0.06)	0.981	583	<0.001
RS144	0.227 (±0.018)	0.274 (±0.024)	−1.19 (±0.07)	0.979	516	<0.001

**Table 2:** Parameters D, G and H of Eq. 2 for rice straw (RS0) and humus-like substances (RS4, RS8, RS24, RS48, RS96, RS144).

Adsorbent	D	G	H	*r* ^2^	F	P
RS0	0.044 (±0.118)	0.217 (±0.160)	−3.11 (±0.43)	0.207	2.86	<0.1
RS4	0.181 (±0.046)	0.183 (±0.062)	−2.08 (±0.17)	0.800	43.9	<0.001
RS8	0.167 (±0.034)	0.196 (±0.047)	−1.97 (±0.12)	0.872	75.2	<0.001
RS24	0.158 (±0.030)	0.179 (±0.041)	−1.72 (±0.11)	0.887	86.1	<0.001
RS48	0.159 (±0.040)	0.188 (±0.054)	−1.66 (±0.14)	0.824	51.4	<0.001
RS96	0.174 (±0.028)	0.176 (±0.038)	−1.49 (±0.10)	0.905	105	<0.001
RS144	0.165 (±0.032)	0.194 (±0.043)	−1.55 (±0.12)	0.886	85.9	<0.001

**Figure 2:**
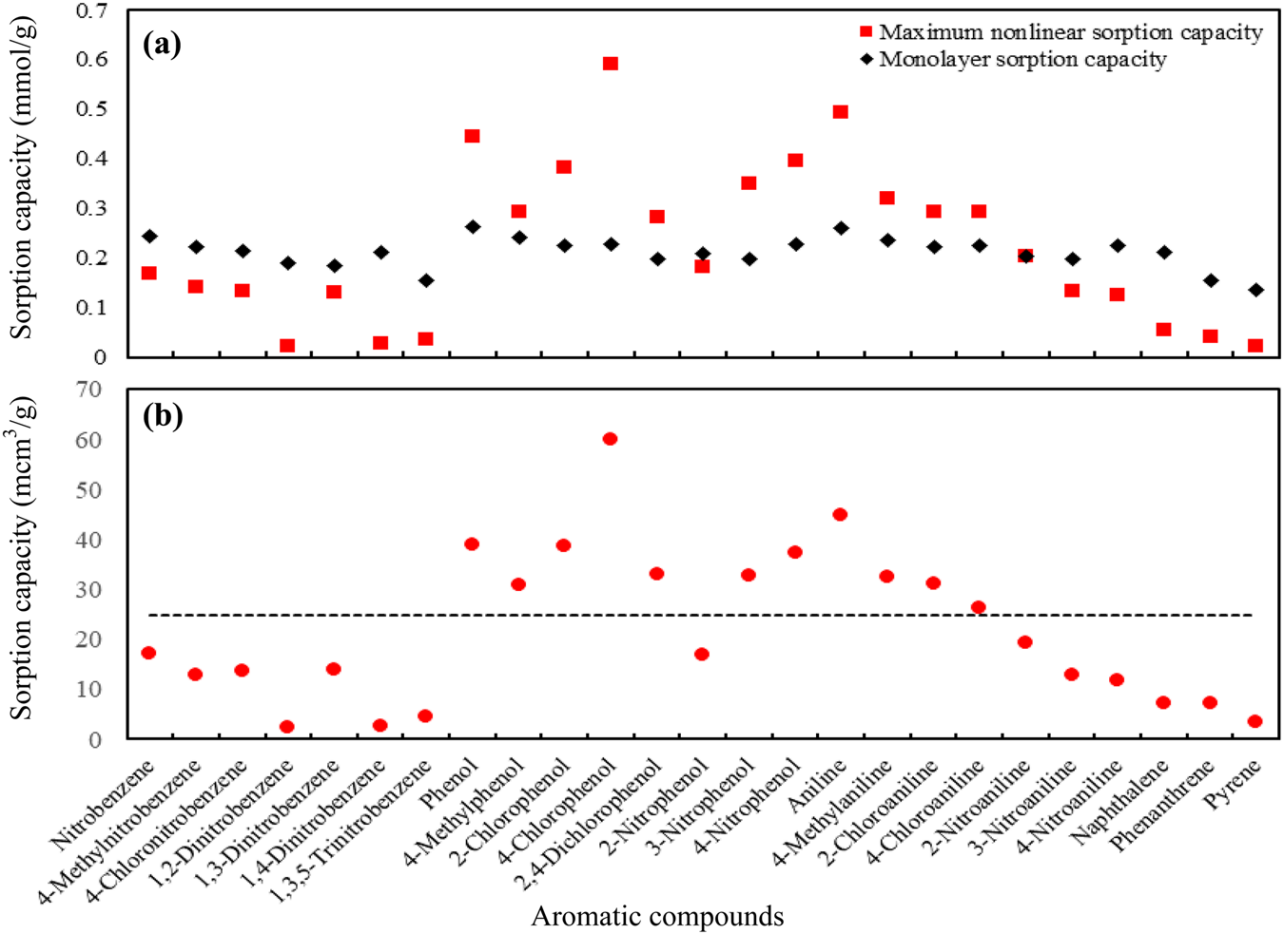
Correlations of nonlinear sorption capacity Q^*^ fitted by DM model with monolayer sorption capacities of 25 aromatic compounds (**a**) and total pore volume (**b**) of RS144. Dotted line indicates total pore volume of humus-like substance.

Adsorption including adsorbing of molecules on surface and filling of molecules into the pores was suggested to interpret the nonlinear sorption by soils and SOM because surface area of these samples were measured in previous studies^32,36,37^. Minerals in soils and HSACM or grassy hard carbon in SOM were suggested as the possible component to give surface area and be responsible for adsorption^5–7,11–13^. Hydrochar, produced from biomass by hydrothermal treatment, was also suggested to give surface area and responsible for adsorption^38^. Surface area, measured by CO_2_ at 273 K, is commonly larger than that measured by N_2_ at 77 K because N_2_ molecules probably fail to access a fraction of pores at such a low temperature (77 K)^21^. Therefore, CO_2_ isotherms is commonly measured and employed to calculate the surface area of SOM and hydrochar for interpreting the adsorption mechanism using larger surface area^21,38^. In this study, surface area (CO_2_) of humus-like substances increased about four times from 20 m^2^/g for RS0 to 82 m^2^/g for RS144 (Table 3). However, the sorption capacity of the nonlinear fraction, i.e., Q^*^ (Table S2), separated from isotherms by DM model fitting, for most of the investigated organic compounds, raised more than four times from that of RS0 to RS144 (Table 3). For example, Q^*^ of 2-NP increased about three orders of magnitude from 0.057 mg/g for RS0 to 25.2 mg/g for RS144 (Table S2). Moreover, Q^*^ of most NOCs, especially for phenols and anilines, by RS144 as an example, was larger than the sorption capacity calculated from the surface area and pore volume of RS144 (Fig. 2). The negatively linear relationship between sorption capacity and molecular size of organic chemicals is the primary characteristic of adsorption mechanism including pore-filling^39^. However, this negative relationship is insignificant between Q^*^ and molecular size (*V*_I_, in Table S3) of organic chemicals (Fig. S10). Therefore, surface area and pore volume of these humus-like substances and the underlying adsorption mechanisms including pore filling failed to account for the observed nonlinear sorption, as is also observed by Jin, *et al*.^40^. In addition, XRD analysis (Fig. S11a,b) showed that the carbon structure of rice straw and humus-like substances was amorphous without hard carbon crystalline because of the absence of well-defined (002) and (10) peaks. These (002) and (10) peaks (Fig. S11b,c) are identified to hard carbons and widely observed for graphite and high temperature treated biochars^41^. Consequently, amorphous carbons are the primary structure of humus-like substances and could be soft carbons and responsible for the nonlinear partition of NOCs^40^.

**Table 3:** Elemental composition and surface area of rice straw (RS0) and humus-like substances (RS4, RS8, RS24, RS48, RS96, RS144)^a^.

Sorbent	C%	H%	N%	O%	Ash%	H/C	O/C	(O+N)/C	*A*_surf_ (m^2^/g)	*V*_toal_ (cm^3^/g)
RS0	41.4 ± 0.6	5.66 ± 0.08	0.573 ± 0.015	41.3	11.1 ± 0.1	1.64	0.748	0.760	20	0.006
RS4	44.0 ± 0.2	5.13 ± 0.04	0.697 ± 0.010	35.6	14.7 ± 0.2	1.40	0.607	0.620	32	0.010
RS8	48.3 ± 0.5	4.95 ± 0.06	0.972 ± 0.005	30.8	15.0 ± 0.2	1.23	0.478	0.495	45	0.014
RS24	50.3 ± 0.1	4.83 ± 0.08	0.949 ± 0.020	28.5	15.4 ± 0.1	1.15	0.425	0.441	59	0.018
RS48	55.8 ± 0.3	4.33 ± 0.03	1.40 ± 0.01	19.0	19.5 ± 0.3	0.931	0.255	0.277	73	0.022
RS96	55.4 ± 0.5	4.30 ± 0.02	1.28 ± 0.02	20.0	19.0 ± 0.1	0.932	0.271	0.291	80	0.024
RS144	56.1 ± 0.6	4.31 ± 0.02	1.32 ± 0.01	19.6	18.7 ± 0.3	0.920	0.261	0.281	82	0.025

^a^H/C, O/C, and (O + N)/C are molar ratios. *A*_surf_ and *V*_toal_ are surface area and total pore volume calculated from CO_2_ isotherms by the nonlocal density fuctional theory (NLDFT).

### Correlations of nonlinear sorption capacity with properties of humus-like substances

**Figure 3:**
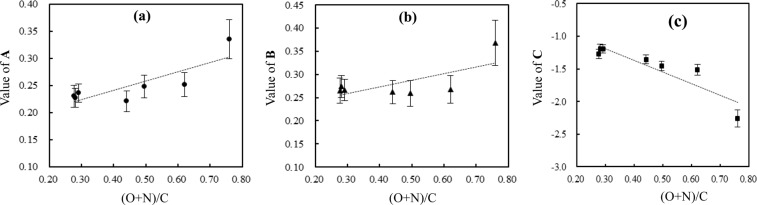
Correlations of parameters A (**a**), B (**b**) and C (**c**) of Eq. 1 with the polarity (O + N)/C of rice straw (RS0) and humus-like substances (RS4, RS8, RS24, RS48, RS96, RS144).

For a given chemical, either DA model fitted nonlinear capacity (i.e., *Q*^0^ in Table S1) or DM model fitted sorption capacity of the nonlinear fraction (i.e., Q^*^ in Table S2), increased with humification degree. For DA model fitted *Q*^0^, positively linear relationships of parameters **A** and **B** of Eq. 1 with the polarity, i.e., (O + N)/C, of rice straw and humus-like substances (Table 3), are obtained (Eqs 4, 5 and Fig. 3a,b), while intercept **C** of Eq. 4 has a negative relationship with the (O + N)/C (Eq. 6, Fig. 3c). For the DM model fitted nonlinear sorption fraction, the parameters **D** and **G** of Eq. 2, could be regarded as constants within the relative errors, i.e., 0.150 (±0.047) and 0.190 (±0.014) respectively (Table 2), while the intercept **H** has negative correlation with the polarity of rice straw and humus-like substances (Eq. 7, Fig. S12a). For the DM model fitted linear sorption fraction, the parameters **J**, **K** and **L** of Eq. 6 also have similar correlations to **A**, **B** and **C** with the polarity (Eqs 8–10, Fig. S12b–d), i.e., **J** and **K** positively correlated with the polarity (Fig. S12b,c), while **L** negatively correlated with the polarity (Fig. S12d). A negative correlation between the polarity and the aromaticity (Table S5) of rice straw and humus-like substances is also observed (Fig. S13), implying that **C**, **H** and **L** are positively correlated with the aromaticity but **A**, **B**, **J** and **K** are negatively correlated with the aromaticity. These observed correlations are consistent with the observations of the partition mechanism, i.e., partition coefficients (*K*_oc_) are negatively related to the polarity but positively correlated with the aromaticity reported in previous works^28,42,43^. With the decrease of the polarity of humus-like substances (Table 3), accompanied by the increase of the aromaticity (Table S5), humus-like substances tend to be more hydrophobic for partitioning of more aromatic molecules.

4 $${\bf{A}}=0.172(\,\pm \,0.053)\times ({\rm{O}}+{\rm{N}})/{\rm{C}}+0.172(\,\pm \,0.026)\,({r}^{2}=0.678,\,{\rm{F}}=10.5,\,{\rm{P}} < 0.05,N=7)$$

5 $${\bf{B}}=0.144(\,\pm \,0.067)\times ({\rm{O}}+{\rm{N}})/{\rm{C}}+0.215(\,\pm \,0.033)\,({r}^{2}=0.477,\,{\rm{F}}=4.56,\,{\rm{P}} < 0.01,\,N=7)$$

6 $${\bf{C}}=-1.791(\,\pm \,0.381)\times ({\rm{O}}+{\rm{N}})/{\rm{C}}-0.652(\,\pm \,0.184)\,({r}^{2}=0.816,\,{\rm{F}}=22.1,\,{\rm{P}} < 0.005,\,N=7)$$

7 $${\bf{H}}=-2.737(\,\pm \,0.534)\times ({\rm{O}}+{\rm{N}})/{\rm{C}}-0.702(\,\pm \,0.259)\,\,({r}^{2}=0.840,\,{\rm{F}}=26.3,\,{\rm{P}} < 0.004,\,N=7)$$

8 $${\bf{J}}=0.113(\,\pm \,0.067)\times ({\rm{O}}+{\rm{N}})/{\rm{C}}+0.262(\,\pm \,0.032)\,({r}^{2}=0.363,\,{\rm{F}}=2.85,\,{\rm{P}} < 0.15,\,N=7)$$

9 $${\bf{K}}=0.143(\,\pm \,0.051)\times ({\rm{O}}+{\rm{N}})/{\rm{C}}+0.194(\,\pm \,0.024)\,({r}^{2}=0.615,\,{\rm{F}}=8.00,\,{\rm{P}} < 0.004,\,N=7)$$

10 $${\bf{L}}=-\,1.857(\,\pm \,0.383)\times ({\rm{O}}+{\rm{N}})/{\rm{C}}-0.573(\,\pm \,0.185)\,({r}^{2}=0.825,\,{\rm{F}}=23.6,\,{\rm{P}} < 0.005,\,N=7)$$

### Correlations of sorption affinity with properties of NOCs and humus-like substances

**Figure 4:**
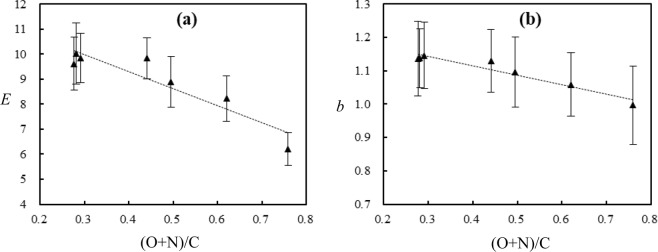
Correlations between **s**orption affinity *E* (**a**), *b* (**b**) of 25 aromatic chemicals and the polarity of rice straw (RS0) and humus-like substances (RS4, RS8, RS24, RS48, RS96, RS144).

DA model fitted *E* and *b* are the parameters that identifying sorption affinity, i.e., the strength of interaction forces between organic molecules and sorbents^18,39^. For rice straw or a given humus-like substance, *E* and *b* values of the investigated NOCs could be considered as a constant within the fitting errors (Fig. S14), indicating that the sorption affinity of NOCs are largely independent of their properties. The average values of sorption affinity *E* and *b* of NOCs by rice straw are closed to 5.71 and 1, respectively (Table S6), coincided with the linear isotherm^18^. However, the average values of *E* and *b* of NOCs by humus-like substances (Table S6) increased with humification degree, presenting more nonlinearity of isotherms by humus-like substances with higher humification degree. Both of *E* and *b* are negatively correlated with the polarity (O + N)/C (Eqs 11, 12 and Fig. 4). These negative correlations indicate that the sorption affinity increased with the aromaticity, as the aromaticity is negatively correlated with the polarity (Fig. S14). Isotherm linearity of NOCs by rice straw should be attributed to the linear partition derived from hydrophobic effect alone^5,35^. Humus-like substances are partitioning medium and the nonlinear partition is responsible for the isotherm nonlinearity of NOCs by humus-like substances, as is indicated by the negative relationships of *Q*^0^, Q^*^ and *Q*_p_ of NOCs with their solubility in water or octanol (Figs 1a,c and S9a). Specific interactions such as hydrogen-bonding interaction could be responsible for the isotherm nonlinearity and nonlinear partition by humus-like substances, as suggested by Spurlock and Biggar^17^ for organic chemicals into SOM, because the chemicals used in this study such as phenols and anilines are with the potential ability to form hydrogen-bonding with humus-like substances. In our recent study^35^, hydrogen-bonding was also observed to be responsible for the nonlinear partitioning of organic molecules. In addition to hydrogen-bonding, the *π*-*π* interaction between the aromatic structure of humus-like substances and organic molecules could be also responsible for the nonlinear partitioning, because nonlinear isotherms were observed for PAHs and nitrobezenes (Figs S1 and S3) by the partitioning medium (i.e., humus-like substances). Therefore, the isotherms of NOCs become more nonlinear, indicated by the increased DA model fitted sorption affinity (*E*), with the increasing aromaticity and the decreasing polarity (O + N)/C of humus-like substances (Eq. 11, Fig. 4). Aromatic structures of humus-like substances not only have the potential to form *π*-*π* interaction with organic molecules but also could act as the hydrogen-bonding acceptor to form hydrogen-bonding interaction with the hydrogen-bonding donors such as phenols and anilines containing –OH and –NH_2_ groups^18,39,44^. With the increasing humification degree, the aromatization degree of aromatic structures of humus-like substances increased (Table S5). Thus, humus-like substances with higher aromatization degree could have the potential to form stronger hydrogen-bonding interaction and *π*-*π* interaction with the organic molecules, resulting in the negative relationships of *E* and *b* with the polarity of humus-like substances, i.e., specific interactions such as hydrogen- bonding interaction and *π*-*π* interaction could be responsible for the nonlinear partition in this study.

11 $${\boldsymbol{E}}=\mbox{--}6.768(\,\pm \,1.242)({\rm{O}}+{\rm{N}})/{\rm{C}}+12.008(\,\pm \,0.602)\,({r}^{2}=0.856,\,{\rm{F}}=29.7,\,{\rm{P}} < 0.003,N=7)$$

12 $${\boldsymbol{b}}=\mbox{--}0.282(\,\pm \,0.036)({\rm{O}}+{\rm{N}})/{\rm{C}}+1.228(\,\pm \,0.017)\,({r}^{2}=0.925,\,{\rm{F}}=61.3,\,{\rm{P}} < 0.001,\,N=7)$$

**Figure 5:**
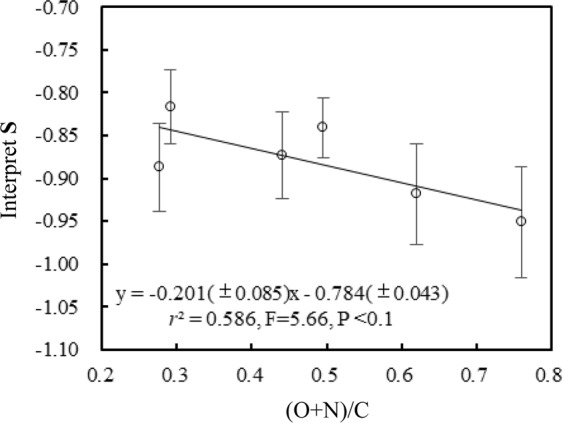
Correlation between interpret **S** of Eq. 13 and the polarity (O + N)/C of humus-like substances (RS4, RS8, RS24, RS48, RS96, RS144).

DM model fitted *K*_*L*_ is also accepted as the affinity coefficient^39^. Isotherms of 25 NOCs sorption by rice straw are almost linear, indicating that nonlinear sorption fraction relative to total sorption is insignificant (Table S2). Therefore, it failed to obtain accurate fitting values of *K*_*L*_ because large deviations of *K*_*L*_ observed for most NOCs and failed to obtain a significant correlation between *K*_*L*_ and the properties of NOCs. However, for humus-like substances, log*K*_*L*_ values of 25 NOCs have negative correlations with their solubility (Eq. 13 and Fig. S15), suggesting that the sorption affinity of nonlinear sorption fraction depends largely on NOCs’ solubility and the capturing of nonlinear partition mechanism.

13 $$\log \,{K}_{L}={\bf{R}}\times \,\log \,{S}_{{\rm{w}}}+{\bf{S}}$$

where **R**, **S** are the coefficient of log*S*_w_ and the intercept, respectively (Table S7). The coefficient **R** is a constant, closed to −0.809 (±0.030) for 6 humus-like substances within the fitting errors, while the intercept **S** of Eq. 16 has negative correlation with the polarity (O + N)/C of humus-like substance (Fig. 5). The negative relationship of **S** with the polarity (Eq. 13 and Fig. 5) is in accordance with that of *E* and *b* with the polarity (Eqs 11, 12 and Fig. 4).

### Conclusion and outlook

Hydrothermal treatment is a reasonable method to prepare humus-like substances with relatively simple structure and homogeneous nature in a short time. The nonlinear sorption of NOCs by humus-like substances observed in this study could be interpreted by nonlinear partition mechanism because of the positive correlation between NOCs’ sorption capacity and their solubility in water and octanol. Decreasing in the polarity of humus-like substances, i.e., increasing the humification degree and aromaticity of humus-like substances, resulted in the enhancement of sorption capacity and affinity of aromatic compounds by humus-like substances. Specific interactions including hydrogen-bonding interaction and π-π interaction of aromatic structures of humus-like substances with organic molecules could be responsible for the nonlinear partition and the increase of sorption affinity. In addition to the positive relationships of sorption capacity with solubility in water and octanol, to make sure the capturing of nonlinear partitioning for sorption by SOM, more characteristics such as whether competitive phenomenon with co-solutes or desorption hysteresis existed or not should be examined for the nonlinear partition mechanism in the future studies. Moreover, other precursors should also be employed to prepare humus-like substances, using hydrothermal treatment under various temperatures, for sorption experiments and the consequent investigation of correlations and mechanisms of nonlinear sorption of NOCs by SOM.

## Materials and Methods

### NOCs

Nitrobenzene (+99%), 1,4-dinitrobenzene (+98%), 4-chloroaniline (+98%) and naphthalene (+99%) were purchased from Acros Organics Co.; 3-Nitroaniline (+98%) was purchased from Sigma-Aldrich Chemistry Co.; 2-Chlorophenol (+99%), 4-chlorophenol (+99%), 2-chloroaniline (+98%), 2-nitroaniline (+99%) and 4-methylaniline (+99.7%) were purchased from Aladdin Reagent Co.; 4-Methylnitrobenzene (+99.5%), 4-chlosronitrobenzene (+98%), phenanthrene (+98%) and pyrene (+97%) were purchased from Fluka Chemistry Co.; 1,2-Dinitrobenzene (+98%), 1,3-dinitro benzene (+99%) and 1,3,5-trinitrobenzene (wetted with 40% water) were purchased from Tokyo Chemical Industry Co.; 2,4-Dichlorophenol (+99.5%), 4-methylphenol (+98%), 2-nitrophenol (+99%), 3-nitrophenol (+99%), 4-nitrophenol (+99.5%) and 4-nitroaniline (+99.5%) were purchased from Sinopharm Chemical Reagent Co.; Aniline (+99.5%) was purchased from Shanghai Reagent Co.; and phenol (+99.7%) was purchased from Hangzhou Shuanglin Chemical Co. All these aromatic chemicals were used as received. The selected properties including water solubility (*S*_w_), octanol-water partitioning coefficients (log*K*_ow_), molecular weight, maximum absorption wavelength (*λ*_max_), dissociated constant (p*K*_a_) of the 25 investigated aromatic chemicals are listed in Table S3.

### Preparation and characterization of rice straw and humus-like substances

Rice straw (RS0) was collected from Zhejiang, China. Before usage, rice straw was cut into 1–2 cm length pieces, washed five times to remove dust and soluble impurities, dried at 75 °C to a constant weight. The humus-like substance samples were prepared from rice straw by hydrothermal method^22^. Briefly, 30 g rice straw pieces and 200 mL deionized distilled (DI) water (Milli-Q, Millipore) were mixed in a 500 mL Teflon-Lined stainless steel autoclave, sealed, and heated at 200 °C for 4, 8, 24, 48, 96 or 144 h to get humus-like substance samples with varied humification degree (i.e., RS4, RS8, RS24, RS48, RS96 and RS144, respectively). The as-prepared samples were washed with DI water five times, dried at 75 °C to a constant weight, and ground to pass a 0.25 mm sieve for usage. The H/C and O/C atomic ratios of the obtained humus-like substance samples are in the range of the atomic ratios of HAs, HMs and soils in the typical Van Krevelen diagram (Fig. S16)^11,15,29,30,40,45^, indicating that hydrothermal treatment is a reasonable method to simulate the humification process.

C, H and N contents of rice straw and humus-like substances were detected by Flash EA 1112 CHN elemental analyzer (Thermo Finningan, USA). Before elemental composition analysis, rice straw and humus-like substances were dried at 105 °C for 8 h. Ash content was measured by weighting the residues after heating the samples in muffle furnace at 850 °C for 4 h. Oxygen content was calculated by mass difference. Solid-state ^13^C NMR spectra was obtained by a Bruker 400 MHz DSX spectrometer (Switzerland) with operating at the ^13^C frequency of 75 MHz. Isotherms of CO_2_ were determined by NOVA-2000E surface area analyzer (Quantachrome, USA) at 273 K. Surface area (*A*_surf_) and total pore volume (*V*_toal_) were calculated by the nonlocal density fuctional theory (NLDFT). Elemental composition and surface area of rice straw and humus-like substances are listed in Table 3. XRD measurement was performed using PANalytical B.V. X-pert Powder system (Nederland) equipped with a Cu monochromator and a MiniProp detector. All scans ran over at 2θ in the range of 5–80°, using a step size of 0.02° and a scan speed of 0.5 s/step.

### Sorption experiments

Isotherms were obtained by a batch equilibration technique at 25 ± 1 °C in 8, 100, or 250 mL glass vials equipped with screw caps, as described in our previous studies^39,46^. Briefly, NOCs, except for naphthalene, phenanthrene, pyrene, 1,2-dinitrobenzene and 1,4-dinitrobenzene, were dissolved in background solution (i.e., DI water containing 0.01 mol/L CaCl_2_ and 200 mg/L NaN_3_ as biocide to avoid biodegradation). Naphthalene, phenanthrene, pyrene, 1,2-dinitrobenzene and 1,4-dinitrobenzene were dissolved in methanol, then diluted with background solution for sorption. The volume fraction of methanol in solution was controlled below 1% to avoid co-solvent effect. The added amounts of rice straw and humus-like substances were controlled to get the percent removal of sorbates over 20%. Solution pH was adjusted by adding 0.1 mol/L HCl or 0.1 mol/L NaOH to suppress the ionization effect of phenols and anilines on sorption. The final solution pH after sorption was about at 8.0 for aniline, 4-methylaniline and 4-chloroaniline, 4.0 for 2-chlorophenol, 2, 4-dichlorophenol, 2-nitrophenol, 3-nitrophenol and 4-nitrophenol, while for other chemicals were at 6.5–7.5. After shaking for 5 days at 150 rpm to reach equilibrium, the mixtures were separated by centrifugation at 3500 rpm for 20 min. Equilibrium concentrations of naphthalene, phenanthrene and pyrene in supernatant were determined by a fluorescence spectrophotometer (Shimadzu, RF-5301PC, Tokyo, Japan) at their excitation (E_x_) and emission (E_m_) wavelengths listed in Table S3. Equilibrium concentrations of other compounds were determined by a UV-spectroscopy (Shimadzu, UV-2450, Japan) at their maximum absorption wavelength (*λ*_max_, Table S3). Experimental uncertainties evaluated using chemical solution only in vials were less than 4% of the initial chemical concentrations, indicating the loss of organic chemicals by such as volatilization and photolysis can be largely ignored. Therefore, sorption amount of aromatic compounds was calculated by the mass difference directly.

### Isotherm fitting

Isotherms were fitted using Polayni theory-based Dubinin-Astakhov (DA) model in Eq. 14 and Dual-Mode (DM) model in Eq. 15.

14 $$\log \,{q}_{{\rm{e}}}=\,\log \,{Q}^{0}-{(\varepsilon /E)}^{b}$$

where, *q*_e_ [mg/g] is the equilibrium solid concentration; *Q*^0^ [mg/g] is the sorption capacity of chemicals; *ε* [kJ/mol], *ε* = −*RT*ln(*C*_e_/*S*_w_) is the effective sorption potential; *R* = 8.314 × 10^−3^ [kJ/mol·K] is the universal gas constant; *T* [K] is the temperature; *S*_w_ [mg/L] is the water solubility; *C*_e_ [mg/L] is the equilibrium aqueous concentration; *E* [kJ/mol] and *b* are parameters that can be used to identify the sorption affinity of 25 aromatic chemicals^36,37^. Freundlich model and linear model, commonly employed to fit the isotherm of NOCs by SOM^27,30^, are two special forms of DA model with *b* = 1 for Freundlich model and with = 5.71, *b* = 1 for linear model^38^.

DM model in Eq. 15 includes a linear partition fraction and a site-limited Langmuir-type nonlinear sorption fraction^9–11^.

15 $${q}_{e}={q}_{p}+{q}_{nL}={K}_{p}{C}_{e}+{Q}^{\ast }\,\cdot \,{K}_{L}{C}_{e}/(1+{K}_{L}{C}_{e})$$

where, *q*_p_ [mg/g] is the linear partition uptake; *q*_nL_ [mg/g] is the nonlinear sorption uptake; *K*_p_ [L/g] is the partition coefficient; Q^*^ [mg/g] is the maximum nonlinear sorption capacity; *K*_*L*_ [mL/g] is the sorption coefficient of Langmuir model. DM model was widely employed to separate the linear and nonlinear fraction of the nonlinear isotherms^9–11^.

All estimated model parameter values and the standard errors were determined by commercial software program (SPSS 22.0). The percent sample deviation (SDEV) was calculated based on the relative error between experimental data and the correlations (Eq. 16) to estimate the fitting goodness.

16 $${\rm{SDEV}}=100\times {\{1/N[{({D}_{{\rm{cal}}}-{D}_{\exp })}^{2}/{{\rm{D}}}_{\exp }^{2}]\}}^{0.5}$$

where, *N* is the number of experimental data points; *D*_exp_ and *D*_cal_ are data from experiment and calculation, respectively.

## Supplementary information

Supplementary Information,
