# Supplementary material for: Correlations and nonlinear partition of nonionic organic compounds by humus-like substances humificated from rice straw
Source: Sci Rep. 2019 Oct 22;9:15131. doi: 10.1038/s41598-019-51406-3 (PMC6805877; doi:10.1038/s41598-019-51406-3)
Supplement: Supplementary file 1 — Supplementary Information, [file 41598_2019_51406_MOESM1_ESM.pdf]

Supplementary Data Cover Sheet

Correlations and nonlinear partition of nonionic organic compounds by humus-like substances humificated from rice straw

Liufen Ren [1, 2, 3], Daohui Lin[1, 2, 3], Kun Yang[*,1, 2, 3]

[1]Department of Environmental Science, Zhejiang University, Hangzhou 310058, China

[2]Key Laboratory of Environmental Pollution and Ecological Health of Ministry of Education, Hangzhou 310058, China

[3]Zhejiang Provincial Key Laboratory of Organic Pollution Process and Control, Hangzhou 310058, China

[*]Corresponding author (Kun Yang). Tel.: 86-571-88982589; Fax: 86-571-88982590; E-mail: kyang@zju.edu.cn

Number of pages: 28

Number of tables: 7

Number of figures: 16

Journal: *Scientific Reports*

Date prepared: July 11, 2019

Table S1. DA model fitted isotherm parameters of 25 aromatic chemicals on rice straw (RS0) and humus-like substances (RS4, RS8, RS24, RS48, RS96, RS144)

Aromatic chemicals	Sorbent	log Q^0 (Q^0, mg/g)	E (KJ/mol)	b	r^2	SDEV %	F	P	N
NB	RS0	1.33±0.05	5.67±0.31	0.890±0.039	0.998	4.05	5.9E+3	<0.001	22
	RS4	1.78±0.02	7.41±0.20	0.974±0.022	0.999	7.25	3.1E+4	<0.001	24
	RS8	1.82±0.02	7.68±0.15	0.984±0.017	1.00	2.49	5.4E+4	<0.001	21
	RS24	1.86±0.01	9.30±0.12	1.15±0.02	1.00	2.20	1.1E+5	<0.001	24
	RS48	1.97±0.01	9.30±0.10	1.12±0.02	1.00	1.55	2.2E+5	<0.001	23
	RS96	2.08±0.02	9.12±0.21	1.11±0.03	0.999	3.39	5.1E+4	<0.001	21
	RS144	2.10±0.02	9.25±0.17	1.12±0.03	0.999	2.94	8.0E+4	<0.001	24
4-MNB	RS0	0.834±0.041	6.22±0.29	0.950±0.047	0.998	3.31	3.7E+3	<0.001	20
	RS4	1.37±0.01	8.56±0.11	1.18±0.02	1.00	1.68	5.1E+4	<0.001	21
	RS8	1.37±0.02	9.25±0.16	1.27±0.04	0.999	2.70	2.7E+4	<0.001	20
	RS24	1.60±0.01	9.45±0.08	1.16±0.02	1.00	1.16	2.6E+5	<0.001	23
	RS48	1.67±0.01	10.1±0.07	1.18±0.01	1.00	1.15	3.1E+5	<0.001	22
	RS96	1.77±0.01	10.0±0.08	1.27±0.02	1.00	1.32	3.0E+5	<0.001	23
	RS144	1.77±0.01	10.2±0.12	1.31±0.02	0.999	2.17	9.2E+4	<0.001	22
4-CNB	RS0	0.332±0.063	6.32±0.42	1.07±0.07	0.995	7.46	3.4E+3	<0.001	20
	RS4	0.984±0.025	7.23±0.17	1.12±0.03	0.999	2.39	1.3E+4	<0.001	20
	RS8	1.04±0.02	7.82±0.12	1.19±0.02	1.00	0.76	2.2E+4	<0.001	20
	RS24	1.24±0.01	8.97±0.09	1.29±0.02	1.00	1.49	5.2E+4	<0.001	21
	RS48	1.24±0.03	9.00±0.25	1.29±0.05	0.998	4.23	6.0E+3	<0.001	19
	RS96	1.32±0.02	9.12±0.12	1.29±0.02	1.00	1.93	3.7E+4	<0.001	21
	RS144	1.44±0.03	8.31±0.22	1.14±0.03	0.999	2.87	1.6E+4	<0.001	20
1,2-DNB	RS0	1.12±0.04	5.97±0.30	0.821±0.035	0.999	1.53	1.6E+4	<0.001	19
	RS4	1.47±0.01	8.69±0.12	1.18±0.02	1.00	1.85	6.7E+4	<0.001	23
	RS8	1.51±0.01	9.11±0.12	1.21±0.02	1.00	2.08	7.1E+4	<0.001	24
	RS24	1.61±0.01	10.1±0.13	1.23±0.02	1.00	2.31	2.6E+4	<0.001	16
	RS48	1.72±0.01	9.68±0.10	1.20±0.02	1.00	1.65	1.6E+5	<0.001	24
	RS96	1.80±0.01	10.1±0.10	1.31±0.02	1.00	2.00	1.3E+5	<0.001	22
	RS144	1.86±0.02	9.99±0.17	1.16±0.03	0.999	2.39	8.1E+4	<0.001	21
		0.834±0.041	6.22±0.29	0.950±0.047	0.998	3.31	3.7E+3	<0.001	20
1,3-DNB	RS0	1.37±0.01	8.56±0.11	1.18±0.02	1.00	1.68	5.1E+4	<0.001	21
	RS4	1.37±0.02	9.25±0.16	1.27±0.04	0.999	2.70	2.7E+4	<0.001	20
	RS8	1.60±0.01	9.45±0.08	1.16±0.02	1.00	1.16	2.6E+5	<0.001	23
	RS24	1.67±0.01	10.1±0.07	1.18±0.01	1.00	1.15	3.1E+5	<0.001	22
	RS48	1.77±0.01	10.0±0.08	1.27±0.02	1.00	1.32	3.0E+5	<0.001	23
	RS96	1.77±0.01	10.2±0.12	1.31±0.02	0.999	2.17	9.2E+4	<0.001	22
	RS144								

Aromatic chemicals	Sorbent	log Q^0 (Q^0, mg/g)	E (KJ/mol)	b	r^2	SDEV %	F	P	N
1,4-DNB	RS0	0.010±0.043	7.38±0.29	1.27±0.07	0.997	5.34	8.0E+3	<0.001	19
	RS4	0.859±0.024	8.05±0.17	1.20±0.03	0.999	2.19	1.4E+4	<0.001	21
	RS8	0.890±0.023	8.62±0.18	1.26±0.03	0.999	2.71	1.1E+4	<0.001	21
	RS24	1.02±0.01	9.31±0.09	1.24±0.01	1.00	1.57	3.9E+4	<0.001	21
	RS48	1.11±0.02	8.54±0.16	1.24±0.03	0.999	2.65	1.1E+4	<0.001	19
	RS96	1.26±0.02	8.81±0.15	1.21±0.02	0.999	2.42	1.8E+4	<0.001	21
	RS144	1.23±0.01	9.32±0.10	1.24±0.02	1.00	1.58	4.4E+4	<0.001	22
TNB	RS0	0.760±0.023	5.57±0.17	0.831±0.017	1.00	2.26	2.2E+4	<0.001	19
	RS4	1.11±0.03	8.51±0.29	0.966±0.032	0.999	3.28	7.5E+3	<0.001	20
	RS8	1.18±0.02	8.69±0.18	1.00±0.03	0.999	2.04	2.3E+4	<0.001	19
	RS24	1.13±0.01	10.6±0.07	1.27±0.02	1.00	1.33	8.0E+4	<0.001	22
	RS48	1.22±0.01	9.41±0.09	1.23±0.02	1.00	1.85	4.6E+4	<0.001	22
	RS96	1.31±0.01	10.2±0.11	1.13±0.02	1.00	1.67	6.8E+4	<0.001	21
	RS144	1.30±0.01	10.2±0.09	1.23±0.02	1.00	1.59	6.8E+4	<0.001	21
Phenol	RS0	2.24±0.03	5.30±0.21	0.922±0.031	0.999	2.09	9.8E+4	<0.001	17
	RS4	2.27±0.03	7.57±0.25	1.06±0.04	0.998	4.39	4.2E+4	<0.001	23
	RS8	2.31±0.03	7.71±0.23	1.05±0.03	0.999	4.16	4.5E+4	<0.001	23
	RS24	2.39±0.03	8.82±0.27	1.06±0.03	0.999	5.36	1.6E+4	<0.001	18
	RS48	2.52±0.04	7.92±0.38	1.02±0.05	0.997	6.20	2.7E+4	<0.001	24
	RS96	2.54±0.03	8.70±0.24	1.08±0.03	0.999	3.92	7.0E+4	<0.001	23
	RS144	2.59±0.03	8.51±0.25	1.03±0.03	0.999	3.65	8.3E+4	<0.001	23
4-MP	RS0	1.88±0.03	6.51±0.18	1.22±0.03	0.999	3.49	2.4E+4	<0.001	20
	RS4	2.09±0.02	7.55±0.16	1.05±0.02	1.00	2.35	9.2E+4	<0.001	22
	RS8	2.07±0.02	8.12±0.16	1.14±0.03	0.999	2.21	1.0E+5	<0.001	21
	RS24	2.12±0.02	10.0±0.23	1.16±0.03	0.999	3.34	5.2E+4	<0.001	22
	RS48	2.24±0.04	8.91±0.33	1.16±0.05	0.998	4.82	3.0E+4	<0.001	21
	RS96	2.40±0.03	8.77±0.27	1.10±0.04	0.998	3.58	6.2E+4	<0.001	20
	RS144	2.41±0.02	8.96±0.23	1.08±0.03	0.999	2.90	1.1E+5	<0.001	22
2-CP	RS0	2.51±0.03	5.39±0.16	0.954±0.021	1.00	1.70	1.9E+5	<0.001	21
	RS4	2.46±0.03	7.67±0.20	1.19±0.03	0.999	3.61	6.0E+4	<0.001	22
	RS8	2.53±0.02	7.65±0.16	1.15±0.03	0.999	2.54	1.4E+5	<0.001	22
	RS24	2.54±0.04	8.85±0.34	1.04±0.04	0.998	4.72	3.7E+4	<0.001	23
	RS48	2.70±0.03	7.98±0.28	0.980±0.030	0.999	2.69	1.4E+5	<0.001	21
	RS96	2.68±0.03	8.82±0.26	1.13±0.03	0.999	3.82	7.7E+4	<0.001	21
	RS144	2.70±0.04	8.89±0.33	1.11±0.04	0.998	4.44	5.4E+4	<0.001	22

Aromatic chemicals	Sorbent	log Q^0 (Q^0, mg/g)	E (KJ/mol)	b	r^2	SDEV %	F	P	N
4-CP	RS0	2.45±0.02	5.53±0.11	0.914±0.01	1.00	2.08	1.4E+5	<0.001	23
	RS4	2.66±0.04	6.47±0.35	0.807±0.03	0.999	2.70	1.2E+5	<0.001	20
	RS8	2.60±0.04	7.52±0.34	0.917±0.04	0.998	4.46	5.2E+4	<0.001	24
	RS24	2.55±0.04	9.25±0.42	1.05±0.04	0.997	6.55	2.2E+4	<0.001	23
	RS48	2.62±0.02	9.35±0.17	1.06±0.02	0.999	2.78	1.5E+5	<0.001	23
	RS96	2.81±0.03	8.57±0.26	0.962±0.029	0.999	3.68	1.1E+5	<0.001	24
	RS144	2.83±0.03	8.60±0.29	0.960±0.031	0.999	3.84	9.4E+4	<0.001	23
2,4-DCP	RS0	2.12±0.03	6.48±0.18	1.01±0.03	0.999	2.70	7.4E+4	<0.001	24
	RS4	2.23±0.03	9.03±0.23	1.12±0.04	0.999	3.67	6.3E+4	<0.001	24
	RS8	2.29±0.03	9.15±0.27	1.09±0.04	0.998	4.27	5.2E+4	<0.001	24
	RS24	2.40±0.04	9.56±0.38	1.04±0.05	0.997	4.90	4.0E+4	<0.001	24
	RS48	2.42±0.04	9.54±0.31	1.27±0.07	0.996	5.29	4.0E+4	<0.001	23
	RS96	2.60±0.03	9.34±0.34	1.06±0.05	0.997	5.00	5.4E+4	<0.001	24
	RS144	2.64±0.04	9.19±0.40	1.02±0.05	0.997	5.35	4.8E+4	<0.001	24
2-NP	RS0	1.38±0.02	5.79±0.10	0.990±0.015	1.00	1.26	6.8E+4	<0.001	20
	RS4	1.69±0.02	7.32±0.15	1.13±0.02	0.999	2.91	3.4E+4	<0.001	24
	RS8	1.76±0.01	7.76±0.12	1.09±0.02	1.00	1.85	1.0E+5	<0.001	23
	RS24	1.87±0.02	9.50±0.17	1.16±0.02	0.999	3.60	1.9E+4	<0.001	17
	RS48	2.01±0.02	8.73±0.15	1.08±0.02	0.999	2.22	1.1E+5	<0.001	23
	RS96	2.05±0.01	9.21±0.12	1.22±0.02	0.999	2.46	1.1E+5	<0.001	25
	RS144	2.11±0.01	9.12±0.13	1.13±0.02	1.00	2.25	1.4E+5	<0.001	24
3-NP	RS0	1.97±0.02	6.26±0.11	1.22±0.02	1.00	2.18	6.5E+4	<0.001	17
	RS4	2.13±0.02	8.34±0.15	1.07±0.02	0.999	2.83	7.9E+4	<0.001	22
	RS8	2.10±0.02	9.38±0.22	1.22±0.04	0.998	4.82	2.8E+4	<0.001	22
	RS24	2.17±0.02	11.3±0.2	1.26±0.03	0.999	4.08	4.9E+4	<0.001	25
	RS48	2.35±0.02	9.95±0.17	1.15±0.02	0.999	3.11	9.7E+4	<0.001	23
	RS96	2.40±0.02	10.3±0.2	1.18±0.03	0.999	4.28	5.4E+4	<0.001	22
	RS144	2.40±0.03	10.4±0.3	1.22±0.05	0.997	6.16	2.8E+4	<0.001	22
4-NP	RS0	2.05±0.03	6.57±0.19	1.08±0.03	0.999	4.07	2.5E+4	<0.001	20
	RS4	2.11±0.02	9.24±0.23	1.18±0.04	0.998	4.73	3.3E+4	<0.001	24
	RS8	2.13±0.03	9.91±0.28	1.22±0.04	0.997	5.87	2.3E+4	<0.001	24
	RS24	2.24±0.02	11.2±0.2	1.18±0.03	0.999	4.21	5.0E+4	<0.001	25
	RS48	2.35±0.03	10.6±0.3	1.21±0.05	0.997	5.95	2.9E+4	<0.001	24
	RS96	2.44±0.02	10.5±0.3	1.17±0.03	0.998	22.94	5.0E+4	<0.001	23
	RS144	2.49±0.02	10.3±0.2	1.09±0.03	0.999	3.94	7.4E+4	<0.001	24

Aromatic chemicals	Sorbent	log Q^0 (Q^0, mg/g)	E (KJ/mol)	b	r^2	SDEV %	F	P	N
Aniline	RS0	1.95±0.03	6.21±0.24	0.929±0.030	0.999	2.44	5.2E+4	<0.001	20
	RS4	2.11±0.02	9.24±0.21	0.939±0.020	0.999	2.26	1.1E+5	<0.001	22
	RS8	2.13±0.02	9.81±0.20	0.936±0.016	1.00	2.27	1.1E+5	<0.001	23
	RS24	2.16±0.02	9.26±0.17	0.954±0.015	1.00	1.83	1.1E+5	<0.001	18
	RS48	2.29±0.01	10.6±0.2	0.974±0.015	1.00	2.19	1.8E+5	<0.001	25
	RS96	2.40±0.03	10.8±0.4	0.995±0.033	0.998	4.76	4.5E+4	<0.001	25
	RS144	2.34±0.02	12.1±0.3	1.07±0.03	0.999	4.16	5.6E+4	<0.001	24
4-MA	RS0	1.64±0.02	7.04±0.17	1.09±0.03	1.00	1.98	4.0E+4	<0.001	17
	RS4	1.83±0.01	9.88±0.06	1.07±0.01	1.00	0.90	4.7E+5	<0.001	21
	RS8	1.90±0.02	10.4±0.2	1.06±0.02	0.999	2.46	7.4E+4	<0.001	21
	RS24	1.93±0.03	10.4±0.3	1.01±0.04	0.998	3.08	4.6E+4	<0.001	19
	RS48	1.92±0.01	11.4±0.1	1.16±0.02	1.00	1.42	2.8E+5	<0.001	21
	RS96	2.10±0.01	11.7±0.1	1.00±0.01	1.00	1.42	3.5E+5	<0.001	22
	RS144	2.05±0.02	12.4±0.2	1.16±0.03	0.999	2.95	9.1E+4	<0.001	23
2-CA	RS0	1.85±0.02	5.50±0.14	0.862±0.01	1.00	2.02	5.7E+4	<0.001	21
	RS4	2.05±0.03	8.09±0.29	0.977±0.03	0.999	4.37	2.8E+4	<0.001	24
	RS8	2.01±0.03	9.03±0.26	1.10±0.04	0.998	4.37	3.1E+4	<0.001	22
	RS24	2.07±0.01	9.90±0.14	1.00±0.02	1.00	1.48	2.5E+5	<0.001	20
	RS48	2.24±0.03	8.98±0.25	1.05±0.03	0.999	4.32	4.0E+4	<0.001	22
	RS96	2.25±0.01	9.93±0.13	1.10±0.02	1.00	1.76	2.6E+5	<0.001	21
	RS144	2.26±0.02	10.2±0.2	1.08±0.02	0.999	2.41	1.5E+5	<0.001	22
4-CA	RS0	1.54±0.02	6.50±0.12	1.05±0.02	1.00	1.66	6.2E+4	<0.001	22
	RS4	1.86±0.02	8.94±0.20	1.02±0.03	0.999	2.00	9.7E+4	<0.001	20
	RS8	1.89±0.01	10.4±0.2	0.959±0.019	1.00	1.40	2.3E+5	<0.001	20
	RS24	1.96±0.01	10.9±0.1	1.02±0.01	1.00	0.68	1.2E+6	<0.001	21
	RS48	2.03±0.02	10.7±0.2	1.15±0.03	0.999	2.12	1.5E+5	<0.001	22
	RS96	2.18±0.02	10.2±0.2	1.08±0.03	0.999	2.32	1.5E+5	<0.001	23
	RS144	2.17±0.01	11.4±0.1	1.07±0.01	1.00	1.55	3.5E+5	<0.001	24
2-NA	RS0	1.22±0.02	6.58±0.16	1.01±0.03	1.00	1.41	3.4E+4	<0.001	17
	RS4	1.67±0.02	8.04±0.19	0.997±0.026	0.999	2.42	5.0E+4	<0.001	22
	RS8	1.69±0.02	8.77±0.14	1.13±0.03	0.999	2.22	7.0E+4	<0.001	21
	RS24	1.76±0.01	10.3±0.1	1.21±0.02	1.00	1.28	2.8E+5	<0.001	22
	RS48	1.86±0.01	9.53±0.08	1.27±0.02	1.00	1.29	2.8E+5	<0.001	21
	RS96	1.95±0.01	9.86±0.12	1.22±0.02	0.999	2.12	1.3E+5	<0.001	21
	RS144	1.96±0.01	9.86±0.13	1.25±0.03	0.999	2.31	1.1E+5	<0.001	22

Aromatic chemicals	Sorbent	log Q^0 (Q^0, mg/g)	E (KJ/mol)	b	r^2	SDEV %	F	P	N
3-NA	RS0	1.12±0.03	6.08±0.17	0.931±0.020	1.00	2.08	1.6E+4	<0.001	21
	RS4	1.49±0.02	8.81±0.18	1.04±0.02	1.00	1.70	5.9E+4	<0.001	22
	RS8	1.56±0.02	9.31±0.18	1.00±0.02	0.999	2.34	4.5E+4	<0.001	24
	RS24	1.62±0.01	10.6±0.1	1.14±0.01	1.00	0.94	3.2E+5	<0.001	21
	RS48	1.75±0.02	10.7±0.2	0.923±0.022	0.999	1.85	9.9E+4	<0.001	21
	RS96	1.75±0.01	10.8±0.1	1.29±0.01	1.00	1.47	2.1E+5	<0.001	24
	RS144	1.73±0.01	11.7±0.1	1.28±0.02	1.00	1.67	1.4E+5	<0.001	21
4-NA	RS0	0.876±0.028	7.94±0.25	1.06±0.03	0.999	3.92	5.6E+3	<0.001	19
	RS4	1.45±0.03	8.09±0.22	1.03±0.03	0.999	3.09	1.8E+4	<0.001	23
	RS8	1.46±0.02	9.21±0.18	1.12±0.03	0.999	2.97	2.6E+4	<0.001	23
	RS24	1.57±0.01	10.1±0.1	1.18±0.01	1.00	1.22	2.0E+5	<0.001	24
	RS48	1.65±0.02	10.2±0.2	1.19±0.03	0.999	2.79	4.4E+4	<0.001	22
	RS96	1.72±0.01	10.6±0.1	1.21±0.03	0.999	2.25	8.0E+4	<0.001	21
	RS144	1.76±0.01	10.5±0.1	1.19±0.01	1.00	1.56	1.8E+5	<0.001	22
Naphthalene	RS0	0.199±0.011	5.30±0.07	1.02±0.02	1.00	2.27	2.1E+4	<0.001	13
	RS4	0.951±0.010	6.37±0.07	1.13±0.02	0.999	2.47	1.6E+4	<0.001	18
	RS8	1.03±0.01	7.03±0.10	1.05±0.02	0.999	3.27	1.2E+4	<0.001	19
	RS24	1.18±0.02	7.82±0.14	1.05±0.03	0.999	2.89	1.3E+4	<0.001	14
	RS48	1.34±0.01	7.33±0.11	1.05±0.03	0.999	3.72	1.8E+4	<0.001	21
	RS96	1.41±0.01	7.90±0.09	1.08±0.02	0.999	2.86	3.1E+4	<0.001	17
	RS144	1.48±0.01	7.97±0.10	1.04±0.03	0.998	2.77	4.0E+4	<0.001	18
Phenanthrene	RS0	0.157±0.027	6.24±0.24	0.888±0.046	0.994	6.84	1.8E+3	<0.001	16
	RS4	0.839±0.010	8.93±0.10	0.959±0.021	0.999	2.15	1.5E+4	<0.001	17
	RS8	0.853±0.008	10.5±0.1	0.939±0.022	0.999	2.26	1.5E+4	<0.001	16
	RS24	1.08±0.01	10.3±0.1	1.05±0.021	0.999	2.57	2.3E+4	<0.001	17
	RS48	1.14±0.01	11.1±0.1	0.953±0.013	0.999	1.44	8.2E+4	<0.001	17
	RS96	1.18±0.01	11.6±0.1	1.02±0.029	0.998	2.70	2.9E+4	<0.001	17
	RS144	1.21±0.01	11.9±0.1	1.08±0.024	0.999	2.33	3.6E+4	<0.001	16
Pyrene	RS0	0.015±0.012	6.75±0.10	0.976±0.021	0.999	2.49	2.0E+4	<0.001	16
	RS4	0.639±0.008	9.62±0.08	1.03±0.02	0.999	1.94	1.0E+4	<0.001	16
	RS8	0.703±0.011	10.4±0.1	1.14±0.03	0.999	2.91	4.9E+3	<0.001	15
	RS24	0.791±0.007	10.8±0.1	1.17±0.02	0.999	1.84	1.8E+4	<0.001	16
	RS48	0.878±0.008	10.8±0.1	1.17±0.02	0.999	2.23	1.7E+4	<0.001	17
	RS96	0.909±0.006	11.2±0.1	1.17±0.02	0.999	1.78	3.3E+4	<0.001	16
	RS144	0.932±0.007	11.3±0.1	1.19±0.02	0.999	1.97	2.7E+4	<0.001	18

Table S2. DM model fitted isotherm parameters of 25 aromatic chemicals on rice straw (RS0) and humus-like substances (RS4, RS8, RS24, RS48, RS96, RS144)

Aromatic chemicals	Sorbent	*K*p (L/g)	Q^* (mg/g)	K_L (L/g)	r^2	SDEV %	F	P	*N*
NB	RS0	9.84E-3±4.53E-5	0.162±0.01	5.9E+44±2E+45	1.000	11.2	4.29E+4	<0.001	22
	RS4	3.19E-2±7.43E-4	5.65±0.58	1.26E-2±2.4E-3	0.999	11.5	2.21E+4	<0.001	24
	RS8	3.48E-2±6.27E-4	7.42±0.55	1.16E-2±1.6E-3	1.000	14.0	3.77E+4	<0.001	21
	RS24	4.66E-2±6.36E-4	14.2±0.48	1.35E-2±8.6E-4	1.000	6.95	8.66E+4	<0.001	24
	RS48	5.51E-2±1.05E-3	19.4±0.80	1.24E-2±9.1E-4	1.000	8.29	4.26E+4	<0.001	23
	RS96	7.52E-2±2.01E-3	21.4±1.53	1.43E-2±2.0E-3	0.999	8.71	1.91E+4	<0.001	21
	RS144	8.20E-2±1.59E-3	20.8±1.10	1.69E-2±1.8E-3	0.999	7.60	3.13E+4	<0.001	24
4-MNB	RS0	2.20E-2±4.64E-4	0.133±0.06	1.02E-1±9.8E-2	0.999	2.19	1.55E+4	<0.001	21
	RS4	6.87E-2±7.42E-3	6.72±1.18	2.91E-2±5.6E-3	0.999	10.0	1.74E+4	<0.001	21
	RS8	9.43E-2±4.07E-3	6.75±0.69	4.57E-2±7.6E-3	0.999	2.05	1.98E+4	<0.001	19
	RS24	1.39E-1±3.84E-3	12.1±0.56	5.23E-2±3.9E-3	0.999	5.32	5.12E+4	<0.001	24
	RS48	1.62E-1±6.23E-3	17.2±0.91	5.21E-2±4.4E-3	0.999	1.99	2.88E+4	<0.001	21
	RS96	2.01E-1±8.71E-3	19.0±1.16	5.87E-2±5.8E-3	0.999	4.65	2.26E+4	<0.001	20
	RS144	2.26E-1±3.50E-3	19.5±0.49	6.71E-2±3.2E-3	1.000	3.18	9.81E+4	<0.001	23
4-CNB	RS0	3.04E-2±3.69E-4	0.113±0.02	2.9E+45±2E+45	0.997	8.28	7.49E+3	<0.001	20
	RS4	1.40E-1±2.23E-3	3.59±0.18	8.89E-2±6.8E-3	1.000	3.44	1.17E+5	<0.001	21
	RS8	1.31E-1±4.11E-3	6.15±0.39	7.08E-2±6.6E-3	1.000	2.78	5.89E+4	<0.001	20
	RS24	2.00E-1±5.17E-3	10.1±0.47	8.50E-2±6.3E-3	1.000	5.68	6.11E+4	<0.001	23
	RS48	2.29E-1±9.99E-3	13.2±0.90	9.73E-2±1.1E-2	0.999	7.48	2.28E+4	<0.001	22
	RS96	2.93E-1±1.15E-2	19.5±1.08	7.78E-2±6.5E-3	0.999	3.59	4.20E+4	<0.001	23
	RS144	2.99E-1±1.44E-2	20.9±1.20	7.79E-2±6.2E-3	0.999	7.53	3.87E+4	<0.001	22
1,2-DNB	RS0	1.55E-2±1.14E-2	0.443±2.42	1.33E-2±5.1E-2	0.996	7.47	4.84E+3	<0.001	20
	RS4	4.83E-2±2.38E-2	4.33±2.96	2.34E-2±1.1E-2	0.999	4.08	2.47E+4	<0.001	20
	RS8	8.94E-2±8.38E-3	2.54±0.64	5.30E-2±1.3E-2	0.999	2.48	3.17E+4	<0.001	20
	RS24	1.64E-1±5.39E-3	4.53±0.31	9.54E-2±7.8E-3	1.000	2.10	8.48E+4	<0.001	21
	RS48	2.08E-1±9.07E-3	2.84±0.43	1.56E-1±3.5E-2	0.999	3.15	1.39E+4	<0.001	19
	RS96	1.99E-1±1.58E-2	5.89±0.85	9.38E-2±1.6E-2	0.999	4.90	2.07E+4	<0.001	21
	RS144	2.72E-1±8.60E-3	3.61±0.39	1.52E-1±2.5E-2	0.999	6.50	2.80E+4	<0.001	20
1,3-DNB	RS0	1.94E-2±1.84E-4	0.28±0.02	8.8E+44±5E+44	0.998	5.76	1.69E+4	<0.001	19
	RS4	6.09E-2±2.43E-3	6.43±0.60	2.54E-2±3.3E-3	0.999	5.20	3.49E+4	<0.001	23
	RS8	6.15E-2±1.78E-3	9.09±0.47	2.39E-2±1.7E-3	1.000	5.92	9.09E+4	<0.001	24
	RS24	9.45E-2±4.49E-3	10.1±0.81	4.97E-2±6.2E-3	0.999	20.8	1.11E+4	<0.001	16
	RS48	1.04E-1±3.20E-3	14.5±0.69	3.31E-2±2.4E-3	1.000	8.90	6.36E+4	<0.001	24
	RS96	1.19E-1±6.98E-3	24.1±1.70	2.58E-2±2.6E-3	0.999	9.58	3.47E+4	<0.001	22
	RS144	1.30E-1±8.28E-3	22.1±1.78	3.50E-2±4.4E-3	0.999	13.6	1.82E+4	<0.001	21

**Aromatic chemicals**	**Sorbent**	***K*p (L/g)**	***Q**** **(mg/g)**	***K_L*** **(L/g)**	r^2	**SDEV %**	**F**	**P**	***N***
1,4-DNB	RS0	1.82E-2±3.47E-3	0.205±0.20	6.72E-2±5.9E-2	0.997	7.92	7.07E+3	<0.001	19
	RS4	1.42E-1±1.06E-2	1.04±0.27	1.89E-1±5.5E-2	0.999	2.30	1.59E+4	<0.001	21
	RS8	1.72E-1±4.59E-3	1.25±0.11	2.29E-1±2.5E-2	1.000	2.86	9.24E+4	<0.001	21
	RS24	1.76E-1±4.37E-3	2.84±0.13	2.32E-1±1.6E-2	1.000	6.84	9.32E+4	<0.001	21
	RS48	1.53E-1±1.37E-2	5.25±0.56	1.00E-1±1.0E-2	1.000	4.91	6.37E+4	<0.001	19
	RS96	2.53E-1±1.75E-2	5.60±0.55	1.56E-1±1.7E-2	0.999	7.02	3.64E+4	<0.001	21
	RS144	2.95E-1±1.01E-2	4.48±0.26	2.34E-1±1.7E-2	1.000	6.69	6.51E+4	<0.001	22
TNB	RS0	1.58E-2±3.24E-4	0.171±0.04	8.08E-2±4.2E-2	0.999	12.6	1.89E+4	<0.001	19
	RS4	5.20E-2±1.26E-3	1.48±0.11	1.58E-1±2.8E-2	0.999	10.5	1.42E+4	<0.001	20
	RS8	5.15E-2±1.93E-3	2.96±0.26	7.01E-2±1.1E-2	0.998	6.50	1.33E+4	<0.001	19
	RS24	5.35E-2±1.83E-3	4.66±0.20	7.65E-2±5.8E-3	0.999	7.34	3.78E+4	<0.001	22
	RS48	5.85E-2±2.14E-3	5.77±0.33	4.54E-2±3.7E-3	0.999	5.54	4.13E+4	<0.001	22
	RS96	7.38E-2±2.86E-3	6.13±0.35	7.73E-2±8.3E-3	0.999	9.54	1.84E+4	<0.001	21
	RS144	6.68E-2±3.58E-3	7.54±0.45	5.73E-2±5.9E-3	0.999	9.57	1.56E+4	<0.001	21
Phenol	RS0	1.86E-3±1.52E-5	-0.176±0.25	2.5E+37±1.6E37	0.999	3.88	1.48E+4	<0.001	17
	RS4	3.03E-3±4.65E-5	6.54±0.73	9.08E-4±2.0E-5	0.995	56.9	4.29E+3	<0.001	23
	RS8	3.12E-3±3.81E-5	13.4±1.04	6.59E-4±1.5E-4	0.999	4.35	2.50E+4	<0.001	23
	RS24	4.33E-3±5.09E-5	19.9±1.20	1.01E-3±1.5E-4	1.000	22.5	2.54E+4	<0.001	18
	RS48	5.37E-3±5.44E-5	20.2±1.20	9.68E-4±1.9E-4	0.999	2.38	3.10E+4	<0.001	24
	RS96	5.75E-3±6.71E-5	39.6±1.70	5.18E-4±5.3E-5	1.000	7.10	5.66E+4	<0.001	23
	RS144	6.11E-3±1.27E-4	41.8±3.13	5.39E-4±9.7E-5	0.999	10.3	1.73E+4	<0.001	23
4-MP	RS0	4.72E-3±5.22E-5	-0.31±0.16	1.3E+45±5E+45	0.998	18.4	7.62E+3	<0.001	20
	RS4	6.46E-3±1.24E-4	9.80±0.95	1.13E-3±2.0E-4	1.000	6.39	4.46E+4	<0.001	22
	RS8	7.16E-3±8.62E-5	11.2±0.62	1.22E-3±1.2E-4	1.000	1.76	9.97E+4	<0.001	21
	RS24	8.99E-3±1.04E-4	19.6±0.60	2.57E-3±1.7E-4	1.000	6.20	5.32E+4	<0.001	22
	RS48	1.25E-2±2.69E-4	15.6±1.45	2.78E-3±6.8E-4	0.999	3.26	1.37E+4	<0.001	21
	RS96	1.53E-2±2.64E-4	26.9±1.72	1.99E-3±2.9E-4	0.999	3.05	2.98E+4	<0.001	20
	RS144	1.54E-2±2.44E-4	31.6±1.56	1.91E-3±2.1E-4	0.999	6.40	4.14E+4	<0.001	22
2-CP	RS0	1.00E-2±4.94E-3	1.95±216	5.81E-5±4.1E-3	0.999	2.02	2.20E+4	<0.001	21
	RS4	1.52E-2±3.82E-4	16.1±3.42	8.78E-4±3.0E-4	0.999	5.11	2.25E+4	<0.001	22
	RS8	1.60E-2±2.98E-4	26.5±3.16	7.05E-4±1.3E-4	1.000	2.32	5.42E+4	<0.001	22
	RS24	1.81E-2±1.82E-4	27.9±1.07	2.79E-3±2.5E-4	1.000	12.6	4.58E+4	<0.001	23
	RS48	2.10E-2±4.30E-4	41.2±3.14	1.61E-3±2.6E-4	0.999	6.59	1.95E+4	<0.001	21
	RS96	2.22E-2±8.07E-4	68.7±7.22	9.66E-4±1.8E-4	0.999	9.56	1.65E+4	<0.001	21
	RS144	2.69E-2±4.72E-4	49.1±3.22	1.89E-3±2.8E-4	0.999	6.09	2.26E+4	<0.001	22

Aromatic chemicals	Sorbent	*K*p (L/g)	Q^* (mg/g)	K_L (L/g)	r^2	SDEV %	F	P	*N*
4-CP	RS0	9.55E-3±4.21E-5	0.984±0.23	1.47E-2±2.1E-2	1.000	5.99	5.71E+4	<0.001	23
	RS4	1.45E-2±9.19E-5	19.5±0.69	2.80E-3±3.0E-4	1.000	1.15	9.20E+4	<0.001	20
	RS8	1.64E-2±1.88E-4	22.9±1.39	3.05E-3±5.5E-4	0.999	3.31	2.38E+4	<0.001	24
	RS24	2.05E-2±2.29E-4	33.0±1.40	3.56E-3±4.3E-4	0.999	13.0	2.88E+4	<0.001	23
	RS48	1.79E-2±3.97E-4	73.5±4.09	1.27E-3±1.5E-4	0.999	12.1	1.75E+4	<0.001	23
	RS96	2.67E-2±5.20E-4	74.4±4.67	1.71E-3±2.5E-4	0.999	11.3	1.58E+4	<0.001	24
	RS144	2.88E-2±6.29E-4	75.8±5.38	1.90E-3±3.2E-4	0.999	11.6	1.21E+4	<0.001	23
2,4-DCP	RS0	3.20E-2±2.07E-4	2.58±0.28	1.10E-2±3.0E-3	1.000	2.93	1.18E+5	<0.001	24
	RS4	5.14E-2±4.31E-4	20.9±0.60	1.02E-2±6.9E-4	1.000	2.78	1.16E+5	<0.001	24
	RS8	5.87E-2±5.96E-4	23.4±0.82	1.26E-2±1.2E-3	1.000	1.99	6.27E+4	<0.001	24
	RS24	7.12E-2±6.61E-4	33.1±0.84	1.51E-2±1.1E-3	1.000	5.06	7.34E+4	<0.001	24
	RS48	9.09E-2±1.17E-3	39.8±1.64	8.93E-3±7.9E-4	1.000	5.35	6.26E+4	<0.001	23
	RS96	1.20E-1±5.31E-4	48.3±0.69	1.50E-2±6.0E-4	1.000	2.47	2.91E+5	<0.001	24
	RS144	1.29E-1±6.29E-4	45.8±0.76	1.86E-2±9.4E-4	1.000	2.39	2.08E+5	<0.001	24
2-NP	RS0	1.14E-2±3.53E-5	0.057±0.02	5.2E+37±3E+45	1.000	3.26	1.20E+5	<0.001	20
	RS4	3.04E-2±3.68E-4	3.17±0.33	8.77E-3±1.4E-3	1.000	2.53	9.38E+4	<0.001	24
	RS8	2.91E-2±7.31E-4	9.51±0.80	5.56E-3±6.3E-4	1.000	4.50	6.27E+4	<0.001	23
	RS24	4.30E-2±1.03E-3	15.4±0.90	1.28E-2±1.3E-3	1.000	23.6	2.25E+4	<0.001	17
	RS48	5.66E-2±1.10E-3	16.1±0.88	1.18E-2±1.2E-3	0.999	5.99	4.08E+4	<0.001	23
	RS96	6.09E-2±2.00E-3	29.6±1.90	7.30E-3±6.8E-4	0.999	8.19	3.59E+4	<0.001	25
	RS144	7.22E-2±1.48E-3	25.2±1.17	1.12E-2±8.8E-4	1.000	10.4	4.89E+4	<0.001	24
3-NP	RS0	8.68E-3±6.63E-5	-0.267±0.21	1.45±5.2E+2	0.999	9.15	2.26E+4	<0.001	17
	RS4	1.12E-2±2.06E-4	17.0±1.19	1.98E-3±2.9E-4	0.999	8.75	2.81E+4	<0.001	22
	RS8	1.27E-2±2.59E-4	20.3±1.34	2.67E-3±4.0E-4	0.999	4.11	1.93E+4	<0.001	22
	RS24	1.66E-2±2.98E-4	35.1±1.21	3.87E-3±3.1E-4	0.999	16.8	2.90E+4	<0.001	25
	RS48	2.06E-2±4.18E-4	44.3±2.07	2.63E-3±2.7E-4	0.999	13.9	2.65E+4	<0.001	23
	RS96	2.48E-2±4.71E-4	52.8±2.24	2.96E-3±3.0E-4	0.999	12.8	2.73E+4	<0.001	22
	RS144	2.72E-2±3.84E-4	48.7±1.70	3.62E-3±3.3E-4	0.999	7.61	3.93E+4	<0.001	22
4-NP	RS0	8.32E-3±1.25E-4	2.18±0.80	1.89E-3±1.5E-3	1.000	6.71	2.82E+4	<0.001	20
	RS4	1.16E-2±1.36E-4	17.2±0.73	2.85E-3±3.1E-4	1.000	2.15	4.70E+4	<0.001	24
	RS8	1.24E-2±1.61E-4	24.6±0.88	2.59E-3±2.2E-4	1.000	5.99	4.97E+4	<0.001	24
	RS24	1.66E-2±2.39E-4	34.4±1.04	4.58E-3±3.5E-4	0.999	14.0	3.49E+4	<0.001	25
	RS48	2.16E-2±6.03E-4	44.2±2.85	3.08E-3±4.9E-4	0.998	12.2	1.25E+4	<0.001	24
	RS96	2.48E-2±3.72E-4	53.8±1.85	3.33E-3±2.9E-4	0.999	12.8	3.58E+4	<0.001	23
	RS144	2.59E-2±5.20E-4	55.2±2.49	3.51E-3±4.2E-4	0.999	17.4	1.91E+4	<0.001	24

Aromatic chemicals	Sorbent	Kp (L/g)	Q^* (mg/g)	K_L (L/g)	r^2	SDEV %	F	P	N
Aniline	RS0	2.16E-3±2.07E-4	7.90±4.34	2.28E-4±1.4E-4	0.998	6.96	8.72E+3	<0.001	20
	RS4	4.23E-3±7.07E-5	16.5±0.70	1.91E-3±2.1E-4	0.999	14.0	2.25E+4	<0.001	22
	RS8	4.62E-3±1.05E-4	19.4±0.92	2.17E-3±2.9E-4	0.998	25.2	1.16E+4	<0.001	23
	RS24	4.49E-3±1.44E-4	21.5±1.57	1.44E-3±2.3E-4	0.998	23.4	6.52E+3	<0.001	18
	RS48	7.06E-3±1.66E-4	32.9±1.41	2.37E-3±2.9E-4	0.998	25.9	1.34E+4	<0.001	25
	RS96	9.98E-3±2.88E-4	39.0±2.26	2.90E-3±5.1E-4	0.997	24.5	7.16E+3	<0.001	25
	RS144	9.23E-3±2.59E-4	46.0±2.08	2.95E-3±4.0E-4	0.998	28.2	8.82E+3	<0.001	24
4-MA	RS0	7.01E-3±5.77E-4	5.56±2.34	1.05E-3±4.5E-4	0.999	3.62	2.00E+4	<0.001	17
	RS4	1.14E-2±4.67E-4	14.6±1.03	4.67E-3±6.3E-4	0.999	21.7	1.45E+4	<0.001	21
	RS8	1.56E-2±2.72E-4	14.0±0.47	9.44E-3±8.0E-4	0.999	13.8	2.90E+4	<0.001	21
	RS24	1.07E-2±9.77E-4	25.3±2.30	3.74E-3±5.9E-4	0.996	16.5	5.40E+3	<0.001	19
	RS48	1.39E-2±6.71E-4	26.8±1.44	5.15E-3±5.8E-4	0.998	16.1	1.08E+4	<0.001	21
	RS96	2.22E-2±7.89E-4	28.7±1.35	1.07E-2±1.4E-3	0.997	22.4	8.68E+3	<0.001	22
	RS144	2.10E-2±8.48E-4	34.2±1.63	8.76E-3±1.1E-3	0.997	18.6	8.14E+3	<0.001	23
2-CA	RS0	1.03E-2±1.69E-4	0.823±0.26	1.49E-2±1.6E-2	0.999	5.21	1.21E+4	<0.001	21
	RS4	2.31E-2±4.71E-4	10.2±0.83	7.37E-3±1.4E-3	0.999	17.7	2.25E+4	<0.001	24
	RS8	2.39E-2±2.83E-4	13.5±0.54	7.61E-3±7.3E-4	1.000	4.87	5.82E+4	<0.001	22
	RS24	2.21E-2±6.03E-4	22.4±1.12	7.11E-3±7.9E-4	0.999	11.7	1.60E+4	<0.001	20
	RS48	3.70E-2±1.03E-3	24.6±2.06	6.87E-3±1.4E-3	0.999	16.3	1.30E+4	<0.001	22
	RS96	3.64E-2±1.17E-3	40.3±2.34	5.22E-3±6.0E-4	0.999	11.3	1.78E+4	<0.001	21
	RS144	3.88E-2±7.85E-4	37.8±1.48	7.05E-3±6.0E-4	0.999	11.1	3.12E+4	<0.001	22
4-CA	RS0	1.37E-2±2.95E-4	1.73±0.44	3.84E-3±1.3E-3	1.000	5.57	6.25E+4	<0.001	22
	RS4	2.64E-2±1.19E-3	14.2±1.21	8.32E-3±1.2E-3	0.998	8.82	1.24E+4	<0.001	20
	RS8	2.95E-2±1.02E-3	15.1±0.83	1.91E-2±2.6E-3	0.997	9.86	8.49E+3	<0.001	20
	RS24	3.31E-2±1.51E-3	24.1±1.41	1.28E-2±1.6E-3	0.997	13.4	1.00E+4	<0.001	21
	RS48	5.07E-2±1.04E-3	25.6±0.87	1.52E-2±1.1E-3	0.999	8.46	3.64E+4	<0.001	22
	RS96	6.83E-2±1.95E-3	30.7±1.61	1.58E-2±1.8E-3	0.999	9.39	1.59E+4	<0.001	23
	RS144	6.14E-2±2.31E-3	37.6±1.81	1.86E-2±2.1E-3	0.998	20.2	1.07E+4	<0.001	24
2-NA	RS0	1.34E-2±3.02E-4	1.03±0.21	9.46E-3±2.7E-03	1.000	1.34	6.21E+4	<0.001	17
	RS4	3.40E-2±1.16E-3	8.62±0.72	1.15E-2±1.4E-03	0.999	9.14	2.98E+4	<0.001	22
	RS8	3.90E-2±1.30E-3	12.0±0.81	1.09E-2±1.1E-03	0.999	6.07	4.31E+4	<0.001	21
	RS24	5.11E-2±1.45E-3	17.4±0.75	1.77E-2±1.4E-03	0.999	8.28	3.70E+4	<0.001	22
	RS48	5.79E-2±3.56E-3	26.0±2.17	9.93E-3±1.1E-03	0.999	6.35	2.40E+4	<0.001	21
	RS96	7.06E-2±2.87E-3	31.3±1.72	1.22E-2±1.0E-03	0.999	7.89	3.36E+4	<0.001	21
	RS144	8.17E-2±3.00E-3	28.0±1.66	1.42E-2±1.4E-03	0.999	5.74	2.70E+4	<0.001	22

Aromatic chemicals	Sorbent	*K*p (L/g)	Q^* (mg/g)	K_L (L/g)	r^2	SDEV %	F	P	*N*
3-NA	RS0	1.41E-2±1.58E-4	0.281±0.04	5.10E-2±1.8E-2	1.000	2.98	3.89E+4	<0.001	21
	RS4	4.08E-2±9.14E-4	4.35±0.24	3.65E-2±3.7E-3	0.999	7.32	3.03E+4	<0.001	22
	RS8	4.52E-2±1.47E-3	6.01±0.42	4.09E-2±6.1E-3	0.998	17.4	1.12E+4	<0.001	24
	RS24	5.70E-2±2.47E-3	10.1±0.65	3.95E-2±5.0E-3	0.998	13.9	1.10E+4	<0.001	21
	RS48	6.51E-2±3.38E-3	11.1±0.78	6.47E-2±1.1E-2	0.995	18.3	4.57E+3	<0.001	21
	RS96	7.58E-2±3.74E-3	19.7±1.15	2.55E-2±2.4E-3	0.999	12.7	2.01E+4	<0.001	24
	RS144	7.31E-2±3.87E-3	18.3±1.03	3.84E-2±4.2E-3	0.998	12.3	9.60E+3	<0.001	21
4-NA	RS0	1.56E-2±2.35E-4	0.605±0.05	6.92E-2±1.4E-2	0.999	2.91	2.90E+4	<0.001	19
	RS4	5.01E-2±1.77E-3	3.85±0.41	3.39E-2±5.9E-3	0.999	11.1	1.81E+4	<0.001	23
	RS8	5.15E-2±2.74E-3	6.84±0.73	2.94E-2±5.1E-3	0.998	12.4	1.11E+4	<0.001	23
	RS24	7.04E-2±2.11E-3	10.1±0.49	4.01E-2±3.4E-3	0.999	13.2	2.61E+4	<0.001	24
	RS48	8.78E-2±3.82E-3	12.0±0.88	4.17E-2±5.6E-3	0.998	12.5	1.25E+4	<0.001	22
	RS96	9.32E-2±4.72E-3	17.3±1.18	3.87E-2±4.9E-3	0.998	10.3	1.04E+4	<0.001	21
	RS144	1.07E-1±3.87E-3	17.1±0.90	4.26E-2±4.1E-3	0.999	13.7	2.02E+4	<0.001	22
Naphthalene	RS0	5.61E-2±7.92E-3	0.269±0.50	0.059±0.099	0.999	6.20	7.18E+3	<0.001	13
	RS4	2.86E-1±1.79E-2	1.02±0.73	0.133±0.118	0.999	5.58	1.88E+4	<0.001	18
	RS8	3.11E-1±1.78E-2	1.79±0.58	0.220±0.111	0.998	2.59	1.02E+4	<0.001	19
	RS24	4.44E-1±1.56E-2	2.74±0.39	0.396±0.103	0.999	3.62	1.26E+4	<0.001	14
	RS48	6.68E-1±1.46E-2	2.91±0.38	0.447±0.127	0.999	3.37	1.97E+4	<0.001	21
	RS96	6.55E-1±1.90E-2	7.19±0.66	0.246±0.040	0.999	2.70	3.15E+4	<0.001	17
	RS144	7.93E-1±1.87E-2	7.13±0.56	0.308±0.046	0.999	1.81	3.80E+4	<0.001	18
Phenanthrene	RS0	0.308±2.82	3.87±28.6	0.234±1.02	0.996	13.1	3.93E+3	<0.001	16
	RS4	4.29±0.157	1.65±0.16	13.2±3.0	0.998	5.11	9.93E+3	<0.001	17
	RS8	3.91±0.145	2.29±0.15	15.7±2.9	0.997	6.49	8.39E+3	<0.001	16
	RS24	5.80±0.332	5.27±0.42	9.72±1.82	0.997	11.4	6.67E+3	<0.001	17
	RS48	7.21±0.212	4.83±0.22	17.5±2.5	0.998	8.07	1.34E+4	<0.001	17
	RS96	7.51±0.480	6.44±0.51	15.3±3.2	0.994	8.74	4.70E+3	<0.001	17
	RS144	7.52±0.418	7.61±0.45	15.4±2.4	0.996	9.33	5.88E+3	<0.001	16
Pyrene	RS0	6.81±0.184	0.132±0.03	53.9±16.9	1.000	5.02	3.24E+4	<0.001	16
	RS4	24.2±0.755	1.41±0.09	114±17	0.999	7.17	1.53E+4	<0.001	16
	RS8	23.4±1.58	2.44±0.18	87.9±12.7	0.998	9.49	8.02E+3	<0.001	15
	RS24	28.1±1.38	3.13±0.16	97.7±10.3	0.998	5.73	1.44E+4	<0.001	16
	RS48	27.2±2.91	4.60±0.36	73.9±10.6	0.997	15.4	7.42E+3	<0.001	17
	RS96	34.3±2.22	4.38±0.26	100±14	0.997	6.37	1.02E+4	<0.001	16
	RS144	37.8±2.25	4.52±0.25	110±13	0.997	11.3	9.20E+3	<0.001	18

Table S3. Selected properties of the investigated aromatic chemicals in this study

Aromatic chemicals	S_w[a]	log K_{ow}[b]	MW[c]	λ_{max}[d]	pK_a[e]	V_I/100[f]	π*[g]	β_m[h]	α_m[i]
Nitrobenzene(NB)	1936	1.85	123.11	269	/	0.631	1.01	0.30	0
4-Methylnitrobenzene(4-MNB)	340	2.37	137.14	284	/	0.729	0.97	0.31	0
4-Chloronitrobenzene(4-CNB)	224.8	2.39	157.56	279	/	0.721	1.11	0.26	0
1,2-Dinitrobenzene(1,2-DNB)	133	1.58	168.11	255	/	0.733	1.42	0.37	0
1,3-Dinitrobenzene(1,3-DNB)	574.9	1.49	168.11	242	/	0.733	1.30	0.46	0
1,4-Dinitrobenzene(1,4-DNB)	69	1.48	168.11	266	/	0.733	1.31	0.46	0
1,3,5-Trinitrobenzene(TNB)	278	1.18	213.1	228	/	0.851	1.63	0.61	0
Phenol	80190	1.46	94.11	269	9.9	0.536	0.72	0.33	0.61
4-Methylphenol(4-MP)	23000	1.92	108.13	276	10.14	0.635	0.68	0.34	0.58
2-Chlorophenol(2-CP)	28500	2.17	128.56	273	8.44	0.625	0.82	0.30	0.72
4-Chlorophenol(4-CP)	26300	2.39	128.56	225	9.38	0.625	0.72	0.23	0.67
2,4-Dichlorophenol(2,4-DCP)	4600	3.23	163	245	7.9	0.720	0.87	0.18	0.78
2-Nitrophenol(2-NP)	2100	1.89	139.11	277	7.17	0.685	1.11	0.37	0.11
3-Nitrophenol(3-NP)	14000	2	139.11	273	8.28	0.685	1.06	0.33	0.82
4-Nitrophenol(4-NP)	16000	1.79	139.11	317	7.15	0.685	1.01	0.32	0.93
Aniline	34160	0.9	93.13	230	4.6	0.567	0.73	0.50	0.16
4-Methylaniline(4-MA)	6640	1.66	107	232	5.1	0.662	0.69	0.51	0.05
2-Chloroaniline(2-CA)	5700	1.9	128.58	232	2.66	0.652	0.83	0.4	0.25
4-Chloroaniline(4-CA)	2755	1.83	128.58	238	4.15	0.653	0.73	0.4	0.31
2-Nitroaniline(2-NA)	1260	1.85	138.13	223	0.29	0.685	1.01	0.46	0.22
3-Nitroaniline(3-NA)	900	1.37	138.13	225	2.5	0.685	0.96	0.46	0.39
4-Nitroaniline(4-NA)	600	1.39	138.13	380	1	0.685	0.91	0.46	0.47
Naphthalene	31.7	3.30	128.18	Ex:218nm; Em:324nm	/	0.753	0.70	0.15	0
Phenanthrene	1.29	4.57	178.23	Ex:250nm; Em:366nm	/	1.015	0.80	0.20	0
Pyrene	0.135	5.13	202.26	Ex:238nm; Em:373nm	/	1.156	0.90	0.25	0

[a]S_w: water solubility (mg/L); [b]K_{ow}: octanol-water partitioning coefficient; [c]**MW**: molecular weight (g/mol); [d]λ_{max}: maximum absorption wavelength (nm); [e]pK_a: dissociated constant; [f]V_I: intrinsic molar volume; [g]π*: polarity/polarizability parameter; [h]β_m: hydrogen-bonding acceptor parameter; [i]α_m: hydrogen-bonding donor parameter. (*Environ. Pollut.* **2016**, *210*, 57

Table S4. Parameters **J**, **K** and **L** of Equation 3 for rice straw (RS0) and humus-like substances (RS4, RS8, RS24, RS48, RS96, RS144)

Adsorbent	**J**	**K**	**L**	r^2	F	P
RS0	0.390(±0.037)	0.334(±0.050)	-2.23(±0.13)	0.962	279	<0.001
RS4	0.289(±0.024)	0.262(±0.032)	-1.49(±0.09)	0.972	385	<0.001
RS8	0.306(±0.019)	0.239(±0.025)	-1.40(±0.07)	0.982	613	<0.001
RS24	0.289(±0.022)	0.241(±0.030)	-1.29(±0.08)	0.974	416	<0.001
RS48	0.301(±0.021)	0.249(±0.029)	-1.24(±0.08)	0.978	485	<0.001
RS96	0.310(±0.019)	0.242(±0.025)	-1.14(±0.07)	0.983	636	<0.001
RS144	0.304(±0.021)	0.243(±0.029)	-1.11(±0.08)	0.977	469	<0.001

Table S5. Structural group analysis of Solid- state ^{13}C NMR spectra of rice straw and humus-like substances [a]

**Adsorbent**	**Distribution of C Chemical Shift(ppm),%**								**$T_{aliphatic\ C}$**	**$T_{aromatic\ C}$**	**Aromaticity**
	**0-50**	**50-60**	**60-96**	**96-108**	**108-145**	**145-162**	**162-190**	**190-220**	**%**	**%**	**%**
RS0	6.40	4.44	63.1	12.9	6.38	2.65	3.25	0.83	86.9	9.03	9.41
RS4	13.5	4.36	48.6	10.3	12.6	5.80	2.91	1.96	76.7	18.4	19.4
RS8	14.9	4.30	47.3	10.4	14.0	6.00	1.77	1.33	76.9	20.0	20.7
RS24	21.4	4.32	32.2	7.39	20.3	8.80	3.06	2.48	65.3	29.1	30.9
RS48	27.4	4.94	19.0	5.32	26.0	10.5	3.64	3.26	56.7	36.4	39.1
RS96	35.1	5.27	3.65	2.63	33.4	12.6	3.88	3.52	46.6	46.0	49.7
RS144	35.3	5.35	2.54	2.16	33.7	12.5	4.57	3.94	45.4	46.1	50.4

[a] Within the 0-220 ppm chemical shift range of NMR spectra, structure carbon assignments are as follows: paraffinic carbons (0-50 ppm), methoxyl carbons (50-60 ppm), carbohydrate carbons (60-96 ppm), anomeric carbons (96-108 ppm), aromatic carbons (108-145 ppm), phenolic groups carbons (145-162 ppm), carboxyl carbons (162-190 ppm), and ketone carbons (190-220 ppm). $T_{aliphatic\ C}$: total aliphatic carbon region (0-108 ppm); $T_{aromatic\ C}$: total aromatic carbon region (108-162 ppm); aromaticity: $T_{aromatic\ C}$ /($T_{aliphatic\ C}$ + $T_{aromatic\ C}$).

Table S6. The average values of DA model fitted sorption affinity *E* and *b* of 25 aromatic chemicals on rice straw (RS0) and humus-like substances (RS4, RS8, RS24, RS48, RS96, RS144)

Adsorbent	*E*	*b*
RS0	6.20(±0.65)	0.997(±0.120)
RS4	8.22(±0.90)	1.06(±0.10)
RS8	8.88(±1.01)	1.10(±0.10)
RS24	9.84(±0.83)	1.13(±0.11)
RS48	9.62(±1.05)	1.14(±0.11)
RS96	9.85(±0.98)	1.15(±0.10)
RS144	10.0(±1.24)	1.14(±0.09)

Table S7. Parameters **R** and **S** of Equation 13 for humus-like substances

Adsorbent	**R**	**S**	r^2	F	P
RS4	-0.814(±0.039)	-0.951(±0.065)	0.951	443	<0.001
RS8	-0.813(±0.035)	-0.918(±0.059)	0.959	532	<0.001
RS24	-0.789(±0.021)	-0.841(±0.035)	0.984	1447	<0.001
RS48	-0.808(±0.030)	-0.873(±0.051)	0.969	709	<0.001
RS96	-0.816(±0.031)	-0.887(±0.051)	0.968	707	<0.001
RS144	-0.815(±0.026)	-0.816(±0.043)	0.977	987	<0.001

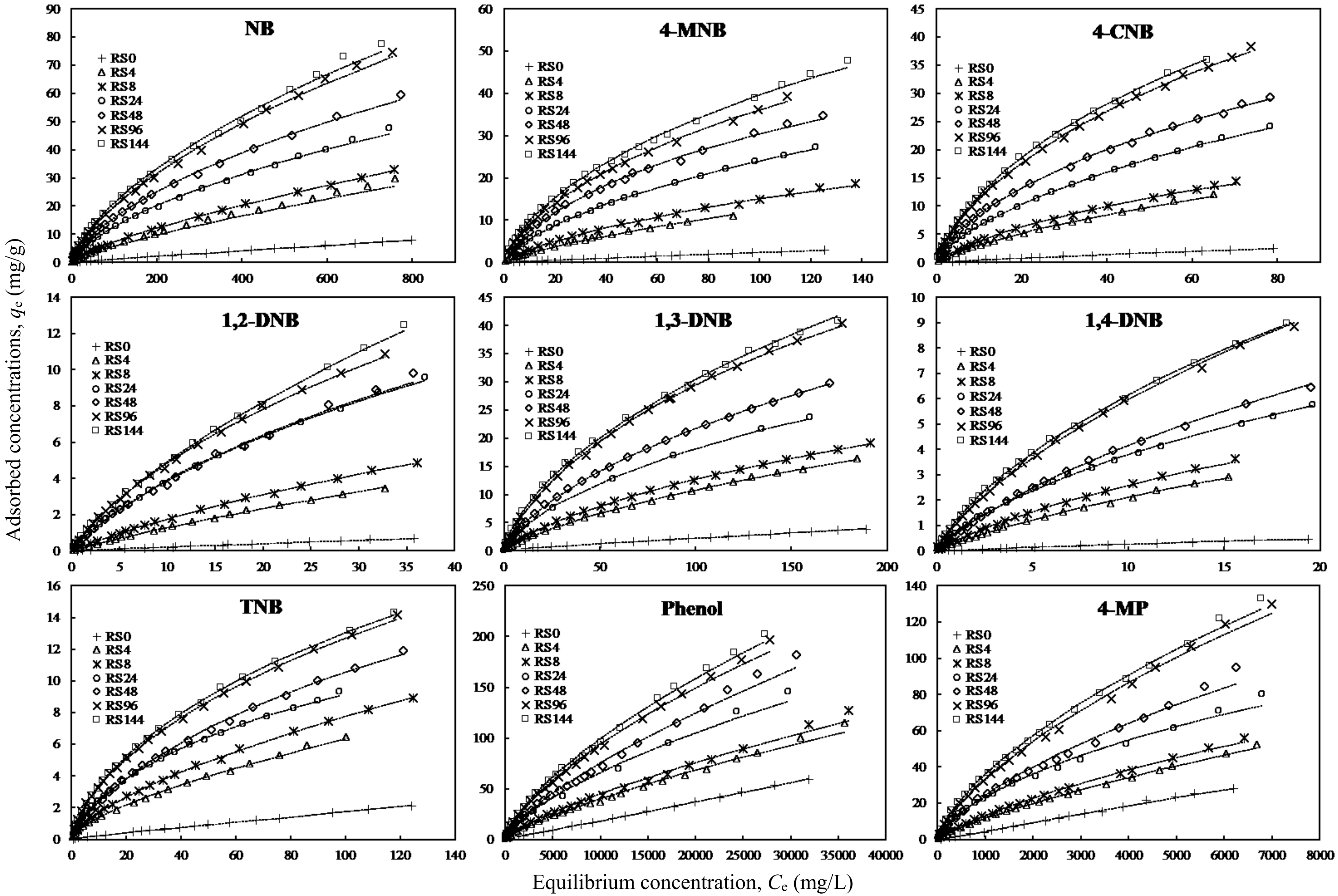

Figure S1. Isotherms and DA model fitting of nitrobenzene, 4-methylnitrobenzene, 4-chloronitrobenzene, 1,2-dinitribenzene, 1,3-dinitribenzene, 1,4-dinitribenzene, 1,3,5-trinitrobenzene, phenol, 4-methylphenol by RS0, RS4, RS8, RS24, RS48, RS96, RS144.

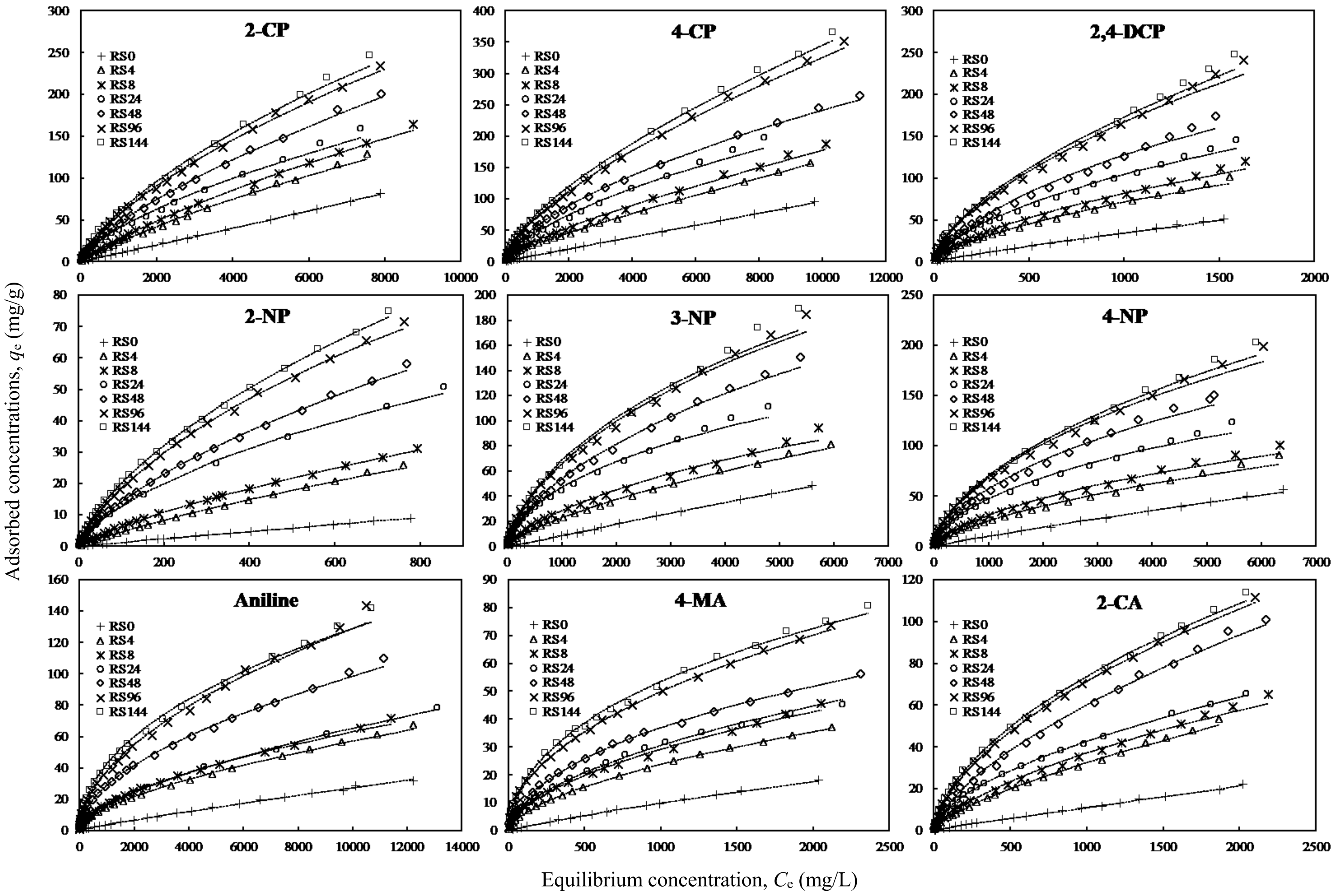

Figure S2. Isotherms and DA model fitting of 2-chlorophenol, 4-chlorophenol, 2,4-dichlorophenol, 2-nitrophenol, 3-nitrophenol, 4-nitrophenol, aniline, 4-methylaniline, 2-chloroaniline by RS0, RS4, RS8, RS24, RS48, RS96, RS144.

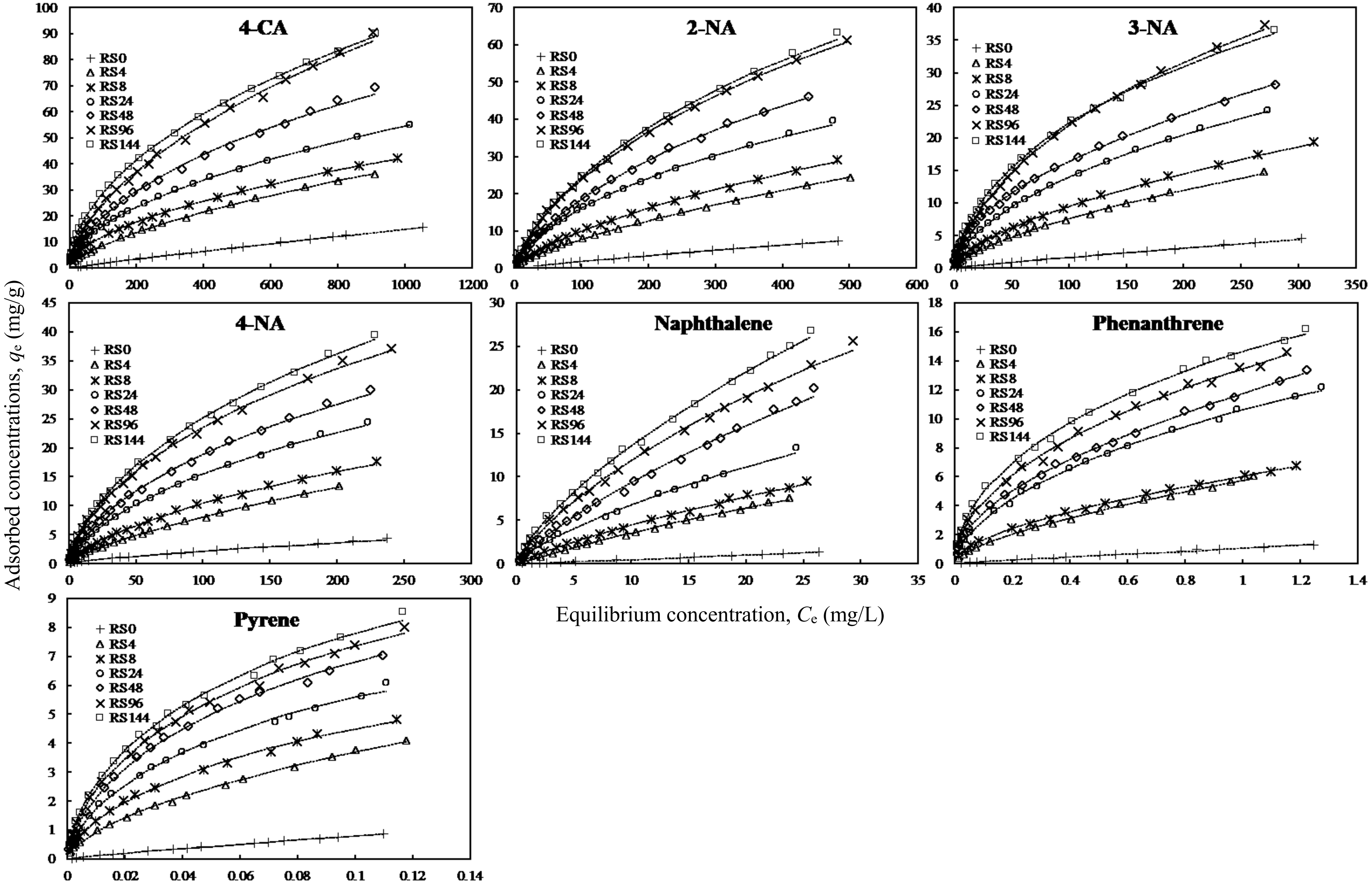

Figure S3. Isotherms and DA model fitting of 4-chloroaniline, 2-nitroaniline, 3-nitroaniline, 4-nitroaniline, naphthalene, phenanthrene, pyrene by RS0, RS4, RS8, RS24, RS48, RS96, RS144.

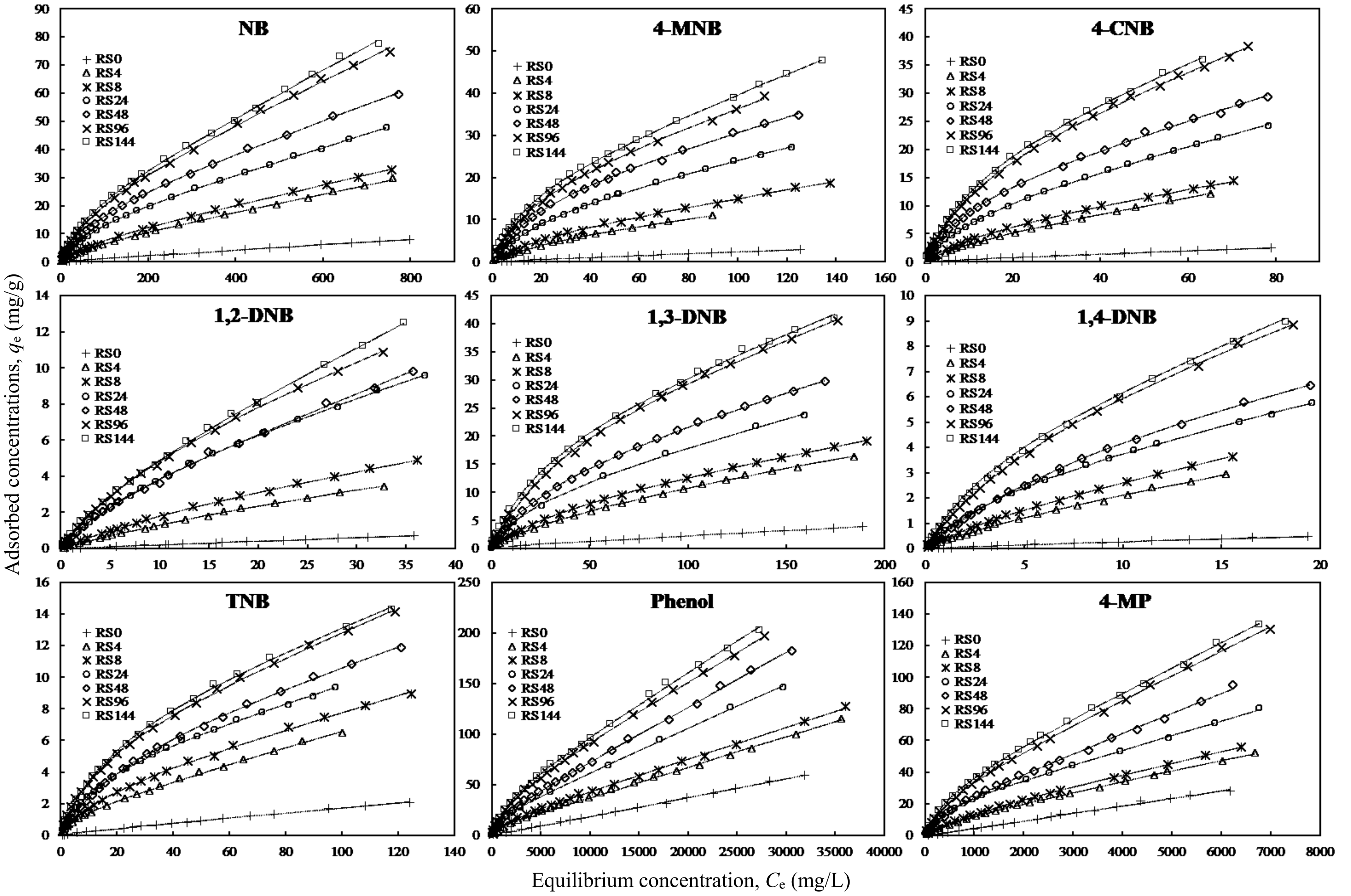

Figure S4. Isotherms and DM model fitting of nitrobenzene, 4-methylnitrobenzene, 4-chloronitrobenzene, 1,2-dinitribenzene, 1,3-dinitribenzene, 1,4-dinitribenzene, 1,3,5-trinitrobenzene, phenol, 4-methylphenol by RS0, RS4, RS8, RS24, RS48, RS96, RS144.

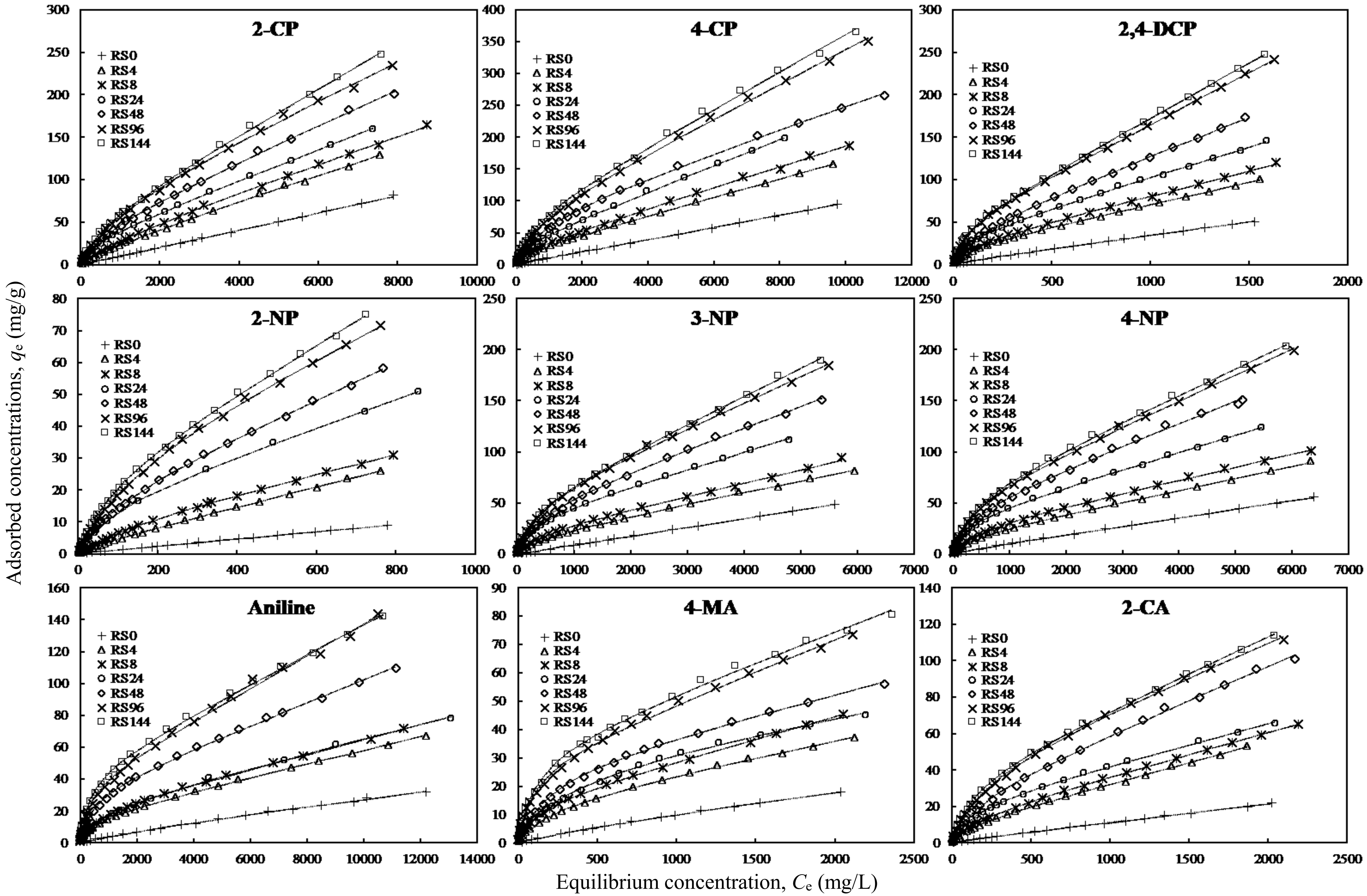

Figure S5. Isotherms and DM model fitting of 2-chlorophenol, 4-chlorophenol, 2,4-dichlorophenol, 2-nitrophenol, 3-nitrophenol, 4-nitrophenol, aniline, 4-methylaniline, 2-chloroaniline by RS0, RS4, RS8, RS24, RS48, RS96, RS144.

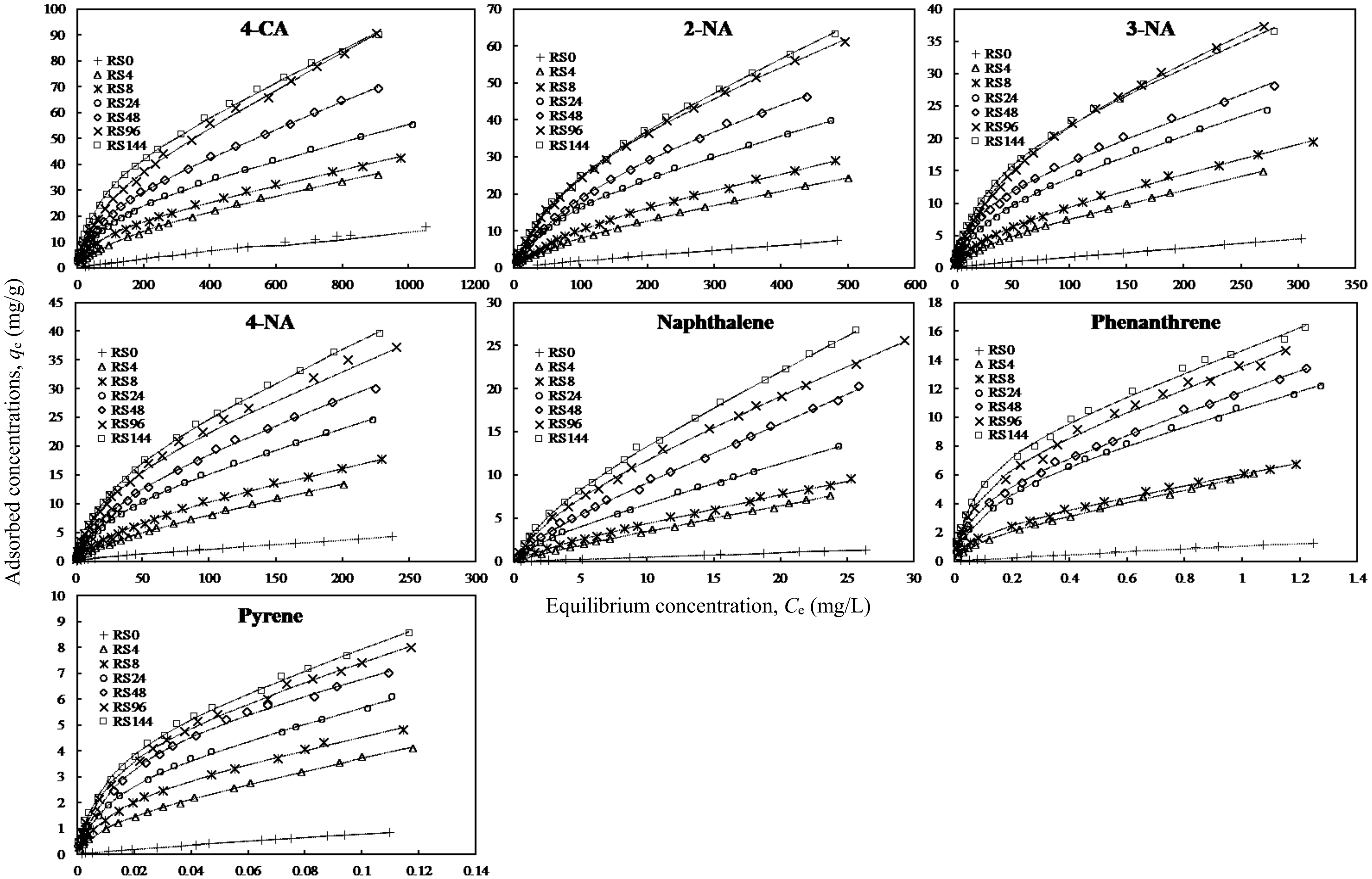

Figure S6. Isotherms and DM model fitting of 4-chloroaniline, 2-nitroaniline, 3-nitroaniline, 4-nitroaniline, naphthalene, phenanthrene, pyrene by RS0, RS4, RS8, RS24, RS48, RS96, RS144.

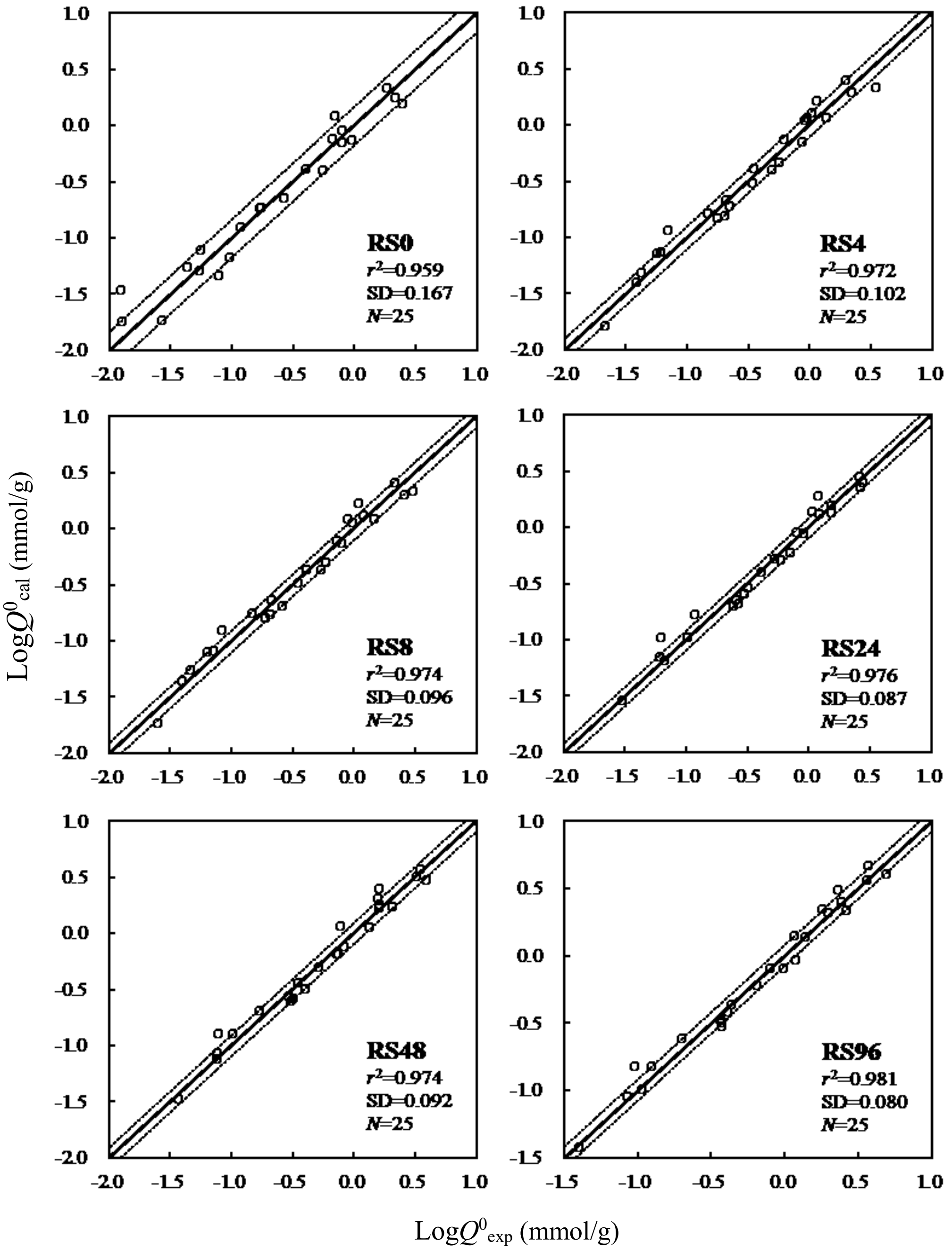

Figure S7. Correlations of calculated logQ^0_{cal} by Equation 1 with logQ^0_{exp} derived from DA model fitted isotherms of NOCs by RS0, RS4, RS8, RS24, RS48, RS96. Solid lines are the reference line, y = x. Dotted lines indicate the standard deviation (SD) values from the reference line.

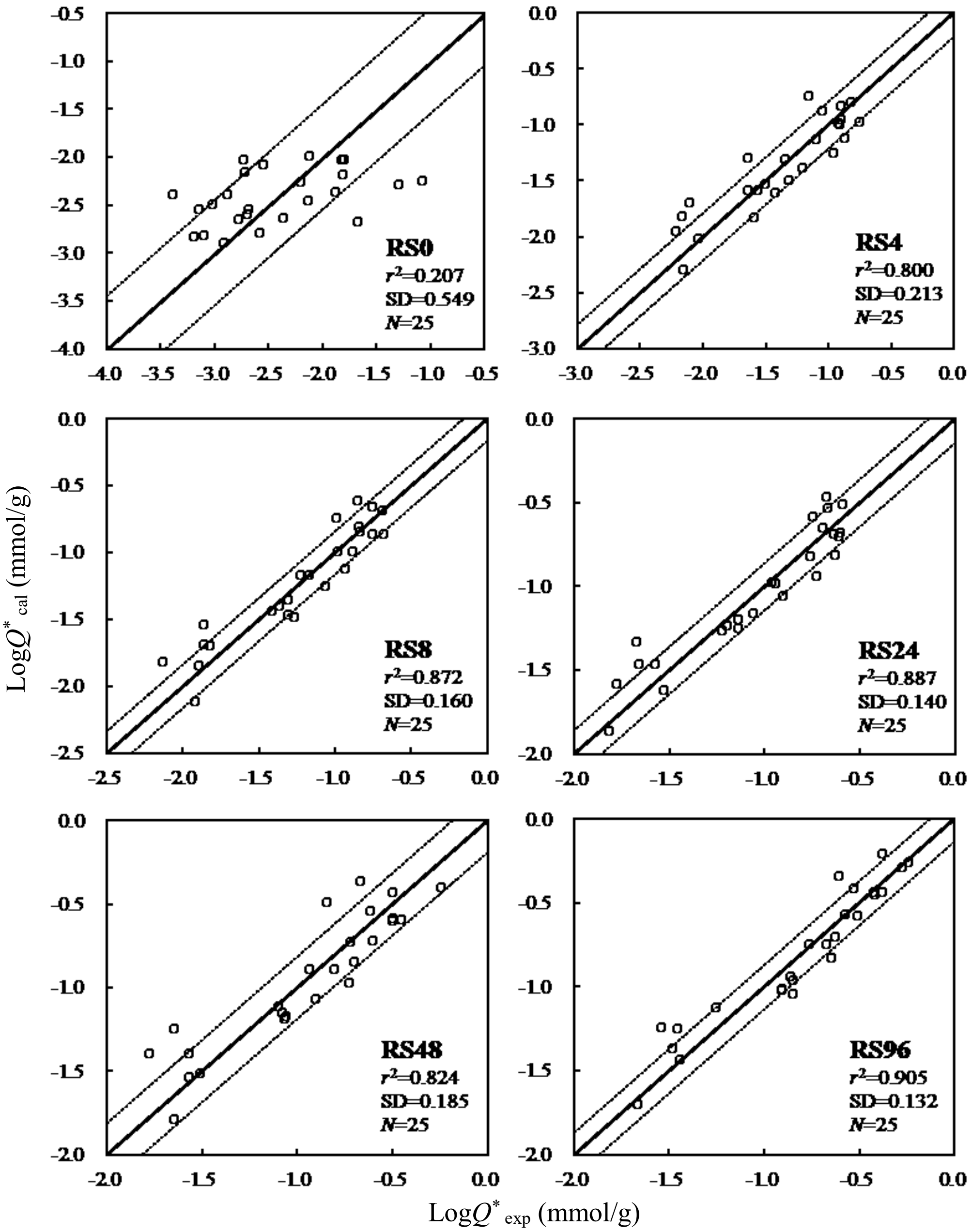

Figure S8. Correlations of calculated logQ^*_{cal} by Equation 2 with logQ^*_{exp} derived from DM model fitted isotherms of NOCs by RS0, RS4, RS8, RS24, RS48, RS96. Solid lines are the reference line, y = x. Dotted lines indicate the standard deviation (SD) values from the reference line.

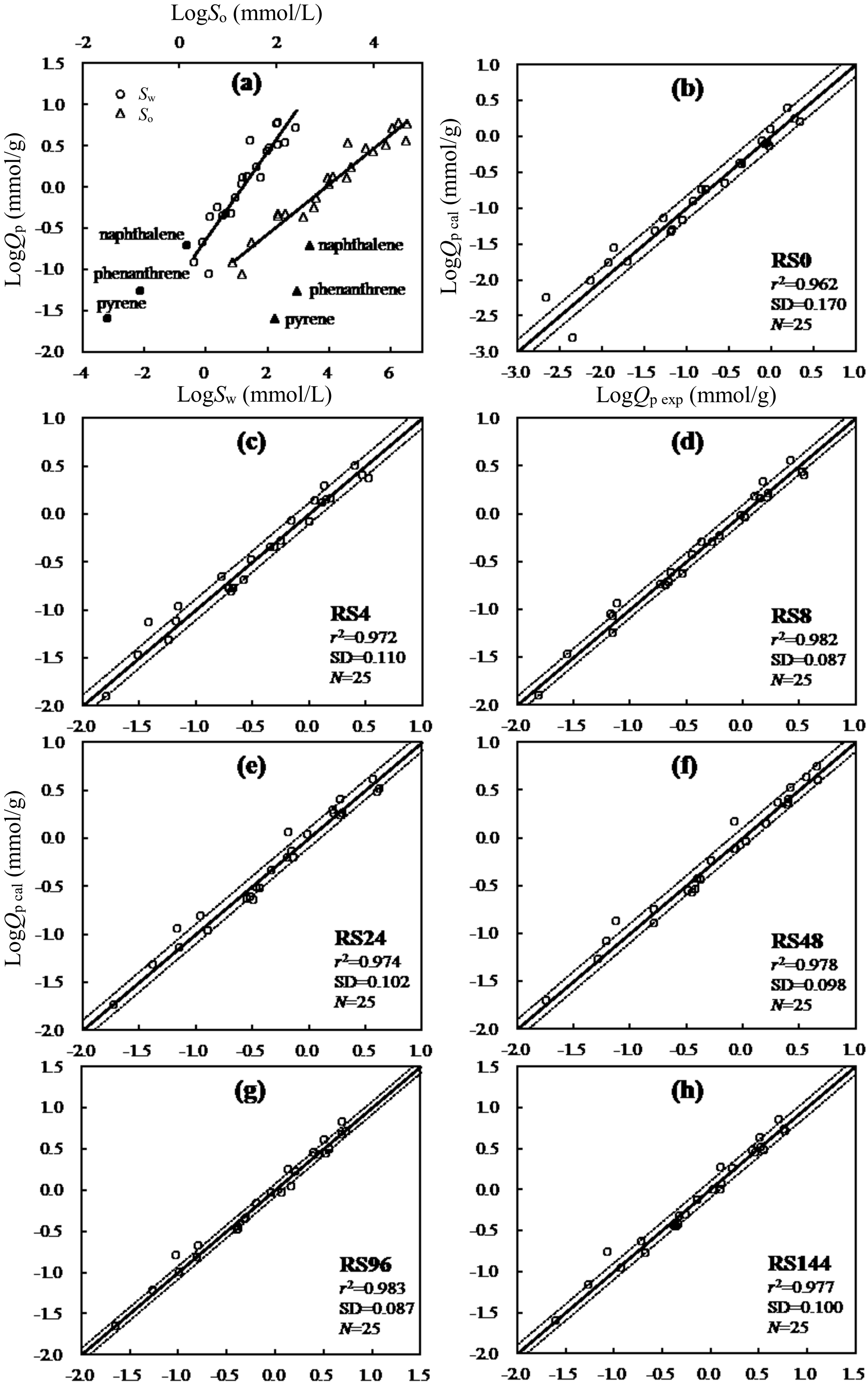

Figure S9. Correlations between logQ_p and logS_w or logS_o of 25 NOCs by RS144 (a) and correlations of calculated log$Q_{p\ cal}$ by Equation 6 with log$Q_{p\ exp}$ derived from DM model fitted isotherms of NOCs by RS0, RS4, RS8, RS24, RS48, RS96, RS144 (b-h). Solid lines are the reference line, y = x. Dotted lines indicate the standard deviation (SD) values from the reference line.

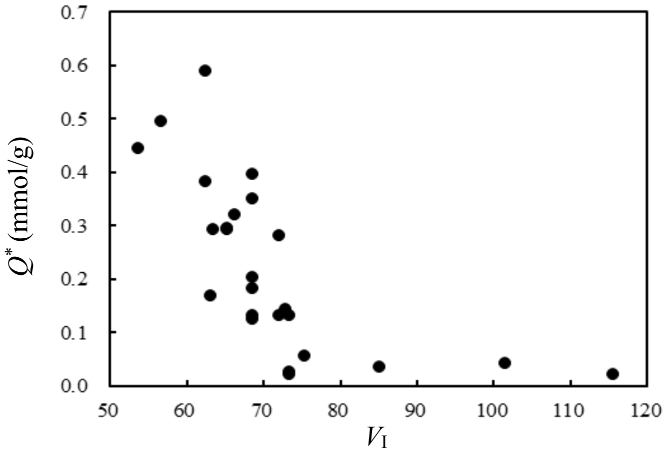

Figure S10. Correlation between Q^* and molecular size (V_I).

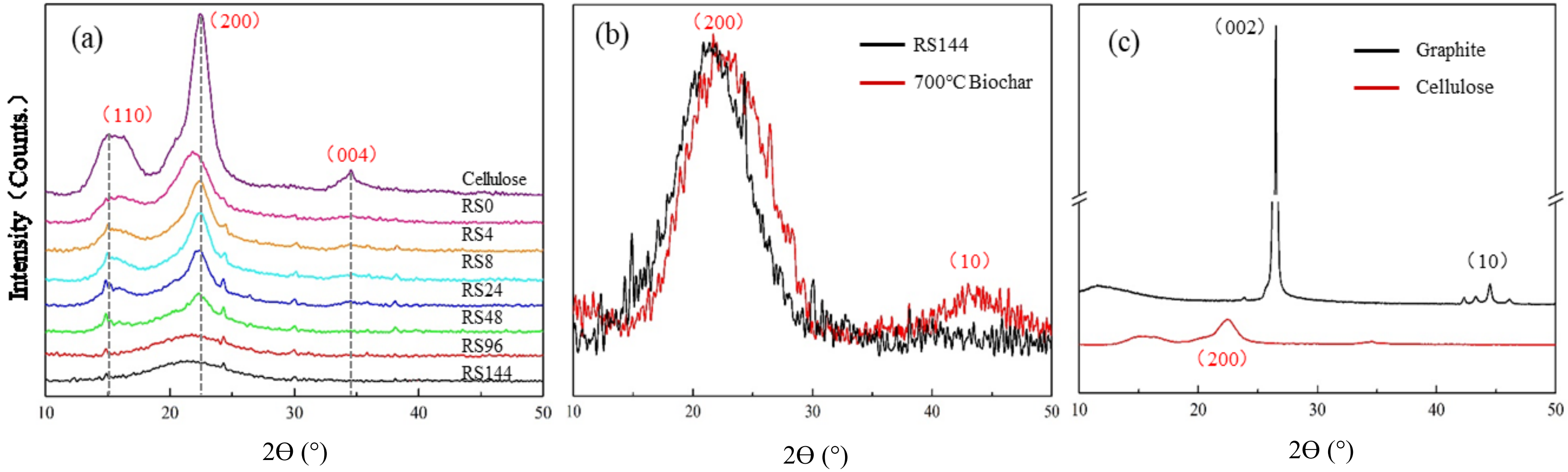

Figure S11. XRD spectra comparison of synthetic graphite, cellulose, 700°C biochar, rice straw and humus-like substances.

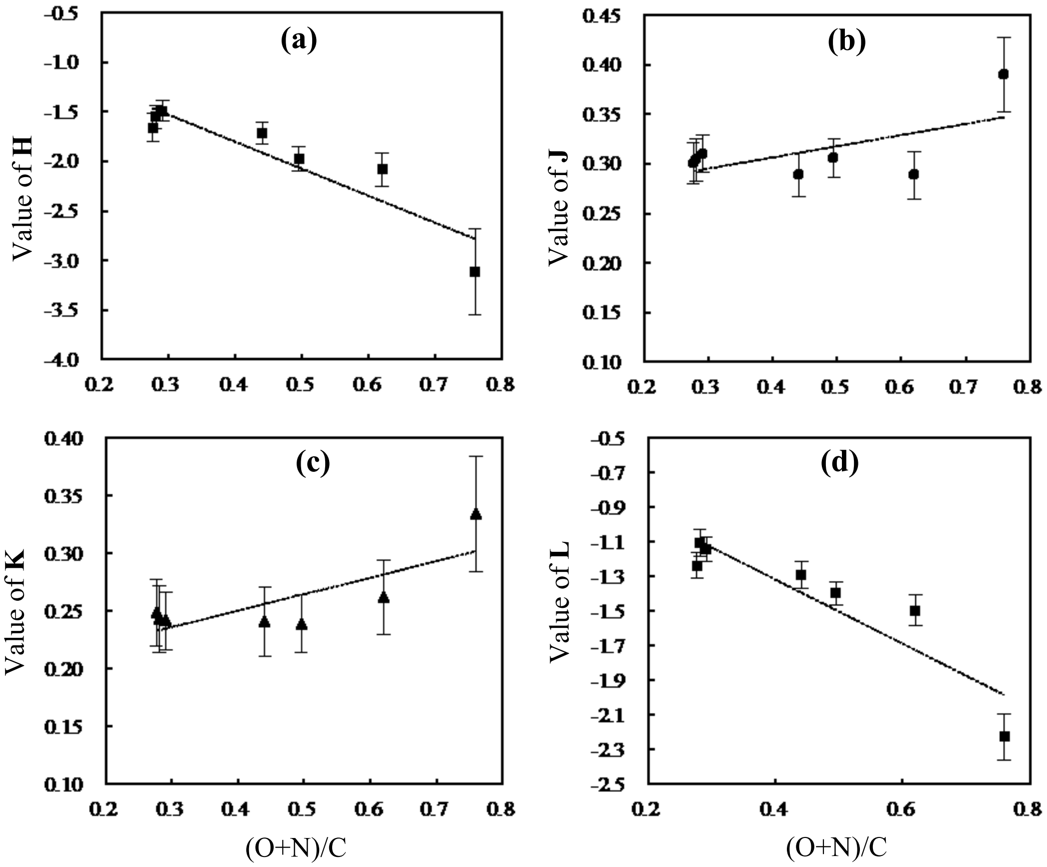

Figure S12. Correlation of **H**, **J, K and L** with the polarity (O+N)/C.

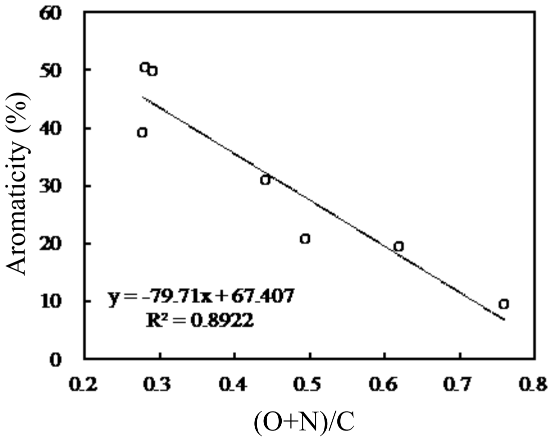

Figure S13. Correlation between the aromaticity (%) and the polarity (O+N)/C.

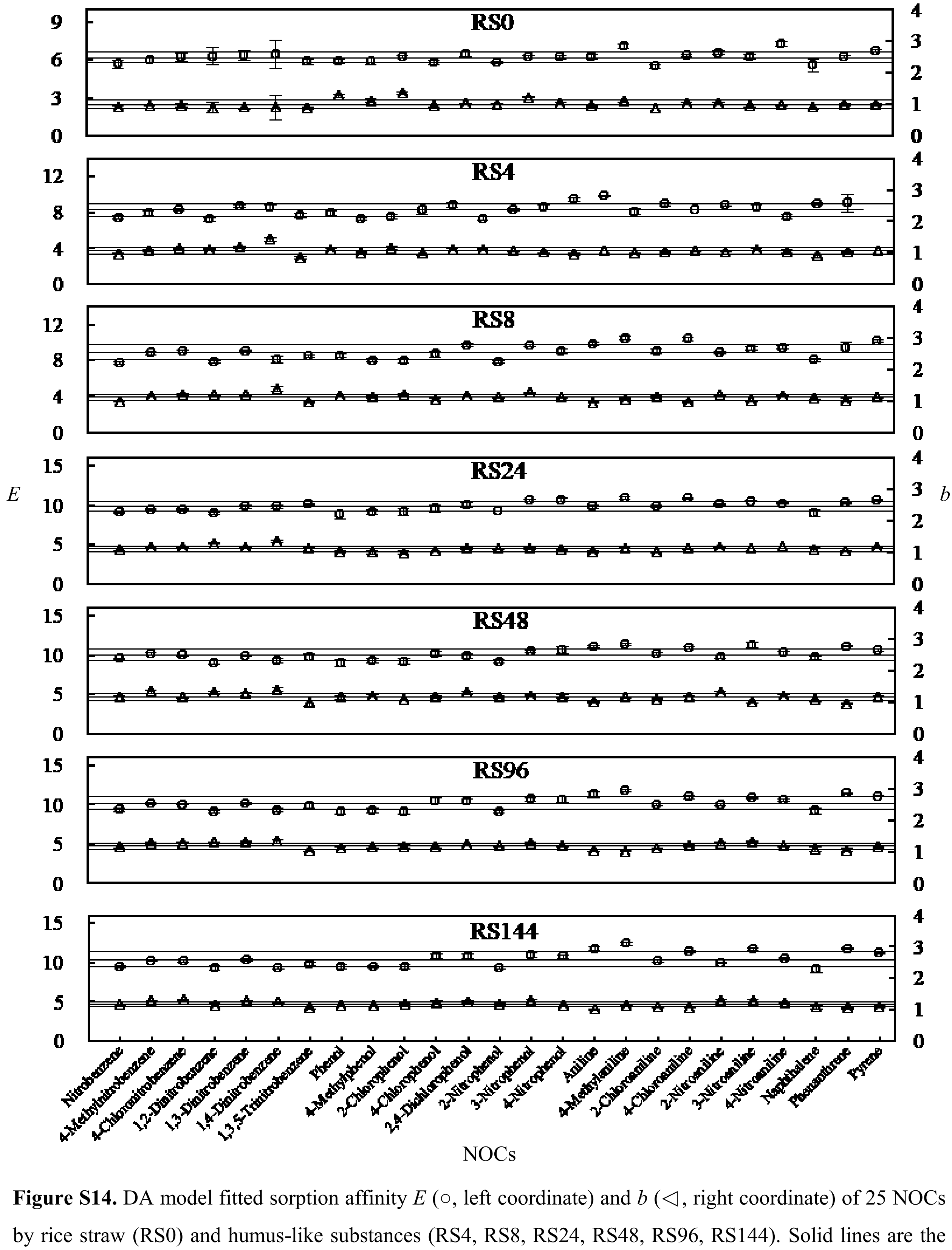

Figure S14. DA model fitted sorption affinity *E* (○, left coordinate) and *b* (◁, right coordinate) of 25 NOCs by rice straw (RS0) and humus-like substances (RS4, RS8, RS24, RS48, RS96, RS144). Solid lines are the average values of *E* and *b*. Dotted lines indicate the standard deviation (SD) values.

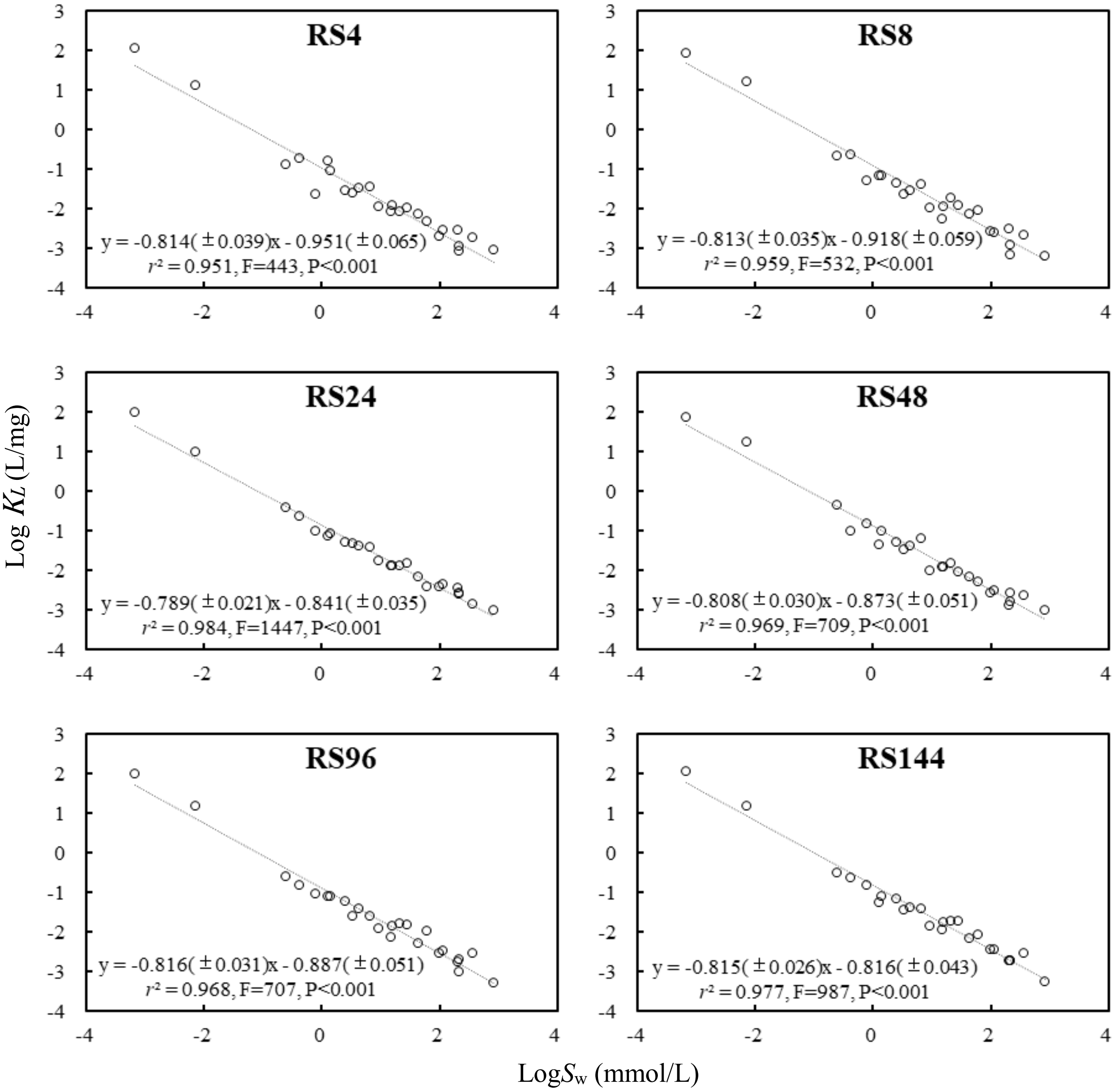

Figure S15. Correlations between logK_L of humus-like substances and logS_w of NOCs.

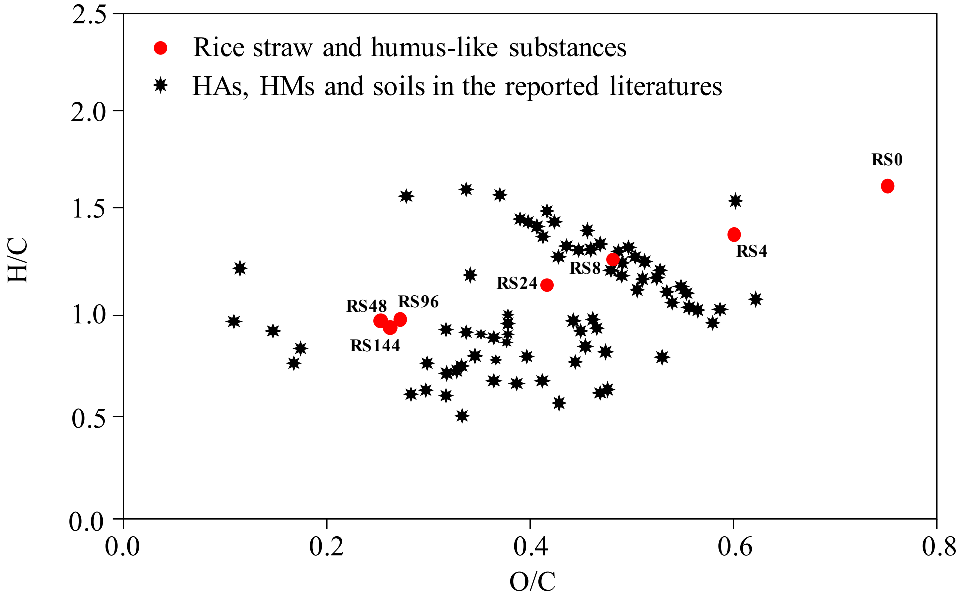

Figure S16. The typical Van Krevelen diagram for H/C vs O/C of rice straw, humus-like substances, HAs, HMs and soils in the reported literatures.
